# Tumor-derived extracellular vesicles as messengers of natural products in cancer treatment

**DOI:** 10.7150/thno.67775

**Published:** 2022-01-16

**Authors:** Yuanxin Xu, Kuanhan Feng, Huacong Zhao, Liuqing Di, Lei Wang, Ruoning Wang

**Affiliations:** 1 College of Pharmacy, Nanjing University of Chinese Medicine, Nanjing 210023, China.; 2 Jiangsu Provincial TCM Engineering Technology Research Center of High Efficient Drug Delivery System, Nanjing 210023, China.; 3 Department of Medicinal Chemistry, School of Pharmacy, China Pharmaceutical University, Nanjing 210009, China.

**Keywords:** Tumor-derived extracellular vesicles, Drug delivery systems, Cancer therapy, Exosomes, Clinical research progress

## Abstract

Extracellular vesicles (EVs) are kinds of two-layer vesicles secreted by cells. They play significant roles in mediating component exchange between cells, signal transduction, and pathological development. Among them, the tumor-derived EVs (TDEVs) are found related to the tumor microenvironment and cancer development. TDEVs can be designed as a natural drug carrier with high tumor targeting and permeability. In recent years, drug delivery systems (DDS) based on TDEVs for cancer treatments have received considerable attention. In this review, the biological characteristics of TDEVs are introduced, especially the effect on the tumor. Furthermore, the various approaches to constructing DDS based on TDEVs are summarized. Then we listed examples of TDEVs successfully constructing treatment systems. The use of chemical drugs, biological drugs, and engineered drugs as encapsulated drugs are respectively introduced, particularly the application progress of active ingredients in traditional Chinese medicine. Finally, this article introduces the latest clinical research progress, especially the marketed preparations and challenges of clinical application of TDEVs.

## Introduction

Extracellular vesicles (EVs) are two-layered vesicles secreted by cells. According to the size and release mechanism, EVs can be divided into exosomes, microvesicles, and apoptotic bodies. As early as the 1960s, when scientists were culturing chondrocytes, they observed that the cells secreted a small vesicle with a diameter of about 100 nm. These small vesicle buds directly from the cell membrane and can induce the formation of hydroxyapatite crystals. Later, researchers collectively referred to the cystic structures released to the outside of cells as "extracellular vesicles." Among them, the name exosomes were first used in the 1980s. A vesicle-like structure substance containing transferrin receptors was discovered during the research process of the maturation of reticulocytes. In 1991, Stein and Luzio named vesicles shed from the membrane of neutrophils as extra nuclear cells. With the gradual deepening of EVs research, people gradually discovered that EVs are not only secreted by certain specific tissues or cells but secreted by all cells. As the winners of the Nobel Prize in Physiology or Medicine in 2013 discovered the regulation mechanism of cellular vesicle transport, in recent years, more and more scientists have devoted themselves to basic research and transformational applications of EVs. This promotes the rapid development of this field 1, 2.

There are still many gaps in our research on the physiological functions of EVs. Early research believed that EVs are trashcans of cell metabolic waste, and their release is conducive to maintaining the stability of the intracellular environment. Recent studies have shown that specifically targeted EVs are a way of signal transduction between cells. EVs also play an important role in a variety of pathological processes, including viral infections, cardiovascular diseases, central nervous system-related diseases, tumor occurrence and development, and immunotherapy. Especially in the development of tumors, tumor-derived EVs (TDEVs) have shown a non-negligible role. In the late 1990s, studies found that exosomes derived from tumor dendritic cells could affect tumor growth. TDEVs can mediate component exchange and signal transduction between tumor cells, promote tumor growth, reshape tumor extracellular matrix, change tumor microenvironment, and regulate tumor development. Therefore, TDEVs have great research value. At present, the related research of EVs is limited by the existing experimental methods, and it is not possible to track the physiological and pathological secretion pathways of EVs *in vivo*. However, the detection of EVs, as one of the liquid biopsy methods, still has great potential for development 3, 4.

Cancer is the number one killer of human health, but there is no effective treatment to overcome this difficulty. Therefore, a large number of researches are aimed at innovating cancer treatment drugs. In 2020, the momentum of innovation in the global tumor treatment field is very strong. A large number of innovative tumor drugs have been approved, behind the booming anti-tumor drug R&D pipeline. Among them, EVs occupy a place as a therapeutic carrier of tumor drugs. A search in this field in Pubmed found more than 1,200 articles related to this field of research. Due to the advantages of EVs such as excellent compatibility, better permeability, natural stability, low immunogenicity, and toxicity, the use of EVs to construct a drug delivery system (DDS) as a carrier for therapeutic drugs has shown significant transformational value. In addition, TDEVs, as a carrier of cancer treatment drugs, have shown their prominence in the field of biomedicine. Based on this, we have also read a lot of relevant literature and found that no one has made a good summary in this field. However, the role of TDEVs in tumor recurrence and metastasis makes its clinical application as a tumor therapy drug-carrier also have certain risks. Therefore, how to design the construction of drug delivery systems to maximize the value of TDEVs is a problem to be solved 5, 6.

In recent years, research on natural products with cancer therapeutic activity has had a very large impact. Traditional Chinese medicines (TCM), including monomeric active ingredients and compound preparations, have been found to have effective therapeutic effects on tumors and have been widely used in the research of tumor therapeutic DDS. A large number of natural products such as traditional Chinese medicine have the characteristics of high hydrophobicity, low solubility, poor stability, and short half-life. As a result, its bioavailability is low and it is difficult to be widely used in clinical practice. At present, many studies have proved that EVs loaded with traditional Chinese medicine ingredients have enhanced efficacy and improved drug resistance. In addition, TDEVs have a better targeting effect than EVs. Therefore, we hope to find a way to construct a TDEVs vector, so that Chinese medicine can be targeted to play its role in the treatment of tumors 7, 8.

This article combines the latest findings of EVs in recent years and reviews the research on TDEVs as tumor therapy drug-carriers. Firstly, the biological characteristics of TDEVs are summarized, especially their biological origin, pharmacokinetics, and their effects on tumors. In addition, the general construction methods of DDS based on EVs are summarized, including separation, drug loading, and engineering modification of EVs. In addition, the application of TDEVs in cancer treatment in recent years is reviewed. It also introduced the promotion of TDEVs in the efficacy of chemical drugs, biological drugs, and engineering drugs, especially the progress in the application of active ingredients in Chinese medicine. Finally, this article introduces the latest clinical research progress, marketing situation, and challenges faced by the clinical application of TDEVs.

## Tumor-Derived Extracellular Vesicles

### Biogenesis of Extracellular Vesicles

#### Biogenesis of Exosomes

**Figure 1:**
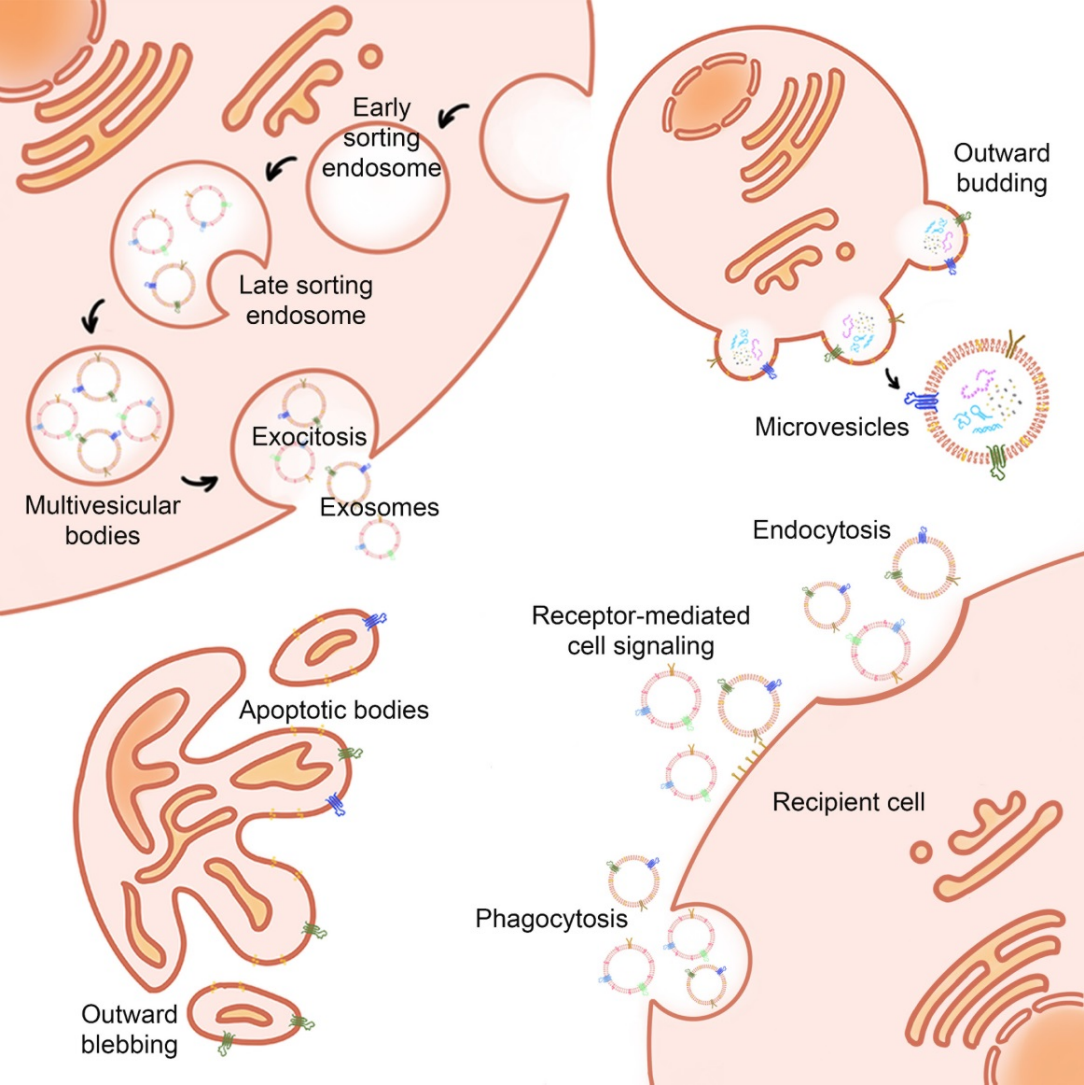
** Biogenesis and uptake of EVs.** EVs are kinds of two-layer vesicles secreted by cells, with diverse bioactive molecules inside, such as proteins, nucleic acids, lipids, and metabolites. The biogenesis of EVs has different ways according to exosomes, microvesicles, and apoptotic bodies. After EVs are distributed *in vivo*, they are finally taken up by target cells. EVs can be internalized by recipient cells in three ways: endocytosis, receptor-mediated cell signaling, and phagocytosis.

Exosomes are EVs in the range of 30-100 nm 9. Exosomes usually occur via the endosome pathway 10. After the inner membrane invaginates into the early endosomes, it is transported and fused to form the late endosomes. Followed by the inner membrane invaginates into the lumen to form the intraluminal vesicles (ILVs), which then mature into multivesicular bodies (MVBs). Eventually, MVBs fuse with the cell membrane, or are degraded by lysosomes, releasing the inner vesicles into the extracellular environment, creating exosomes 11 (**Figure 1**).

Specifically, the first step in exosome formation is the transformation of endosomes into MVBs. One of the key steps is the formation of ILVs in the late endosomes, which is mainly controlled by the mechanism of the endosomal sorting complex required for transport (ESCRTs) 12. ESCRTs protein complexes consist of ESCRT-0, ESCRT-Ⅰ, ESCRT-Ⅱ, ESCRT-Ⅲ, and Vps4 complexes, each containing several subunits and about 30 proteins. In the process of MVBs formation activated by ESCRT, the ESCRT-0 identifies and internalizes the ubiquitinylated proteins through the recruiting proteins at first. Followed by the ESCRT-Ⅰ and ESCRT-Ⅱ help the plasma membrane endothelium to form vesicles, and then ESCRT-Ⅱ activates ESCRT-Ⅲ to bind to the neck of vesicles, actuates the separation of the plasma membrane. Finally, the complex Vps4 was involved in the rupture of the plasma membrane, forming ILVs, which resulted in the evolution of late endosomes into MVBs 13.

In addition, there are some mechanisms that are not associated with ESCRTs, especially under hypoxic conditions in the tumor microenvironment 14, 15. Among them, the tetraspanins superfamily, ceramide, and small GTPases in the Rab family are all involved in the generation of ILVs 16. Tetraspanins aggregate related molecules in the region where ILVs are likely to form, causing the membrane to invaginate. CD9, CD63, and CD81 have been shown to be involved in vesicle transport, and the extra-membrane CD63-rich domain contributes to the formation of ILVs as well 17. In addition, ceramide can induce plasma membrane budding, and small GTPases are involved in vesicle transport and plasma membrane fusion, suggesting that both of these proteins promote the production of exosomes 18.

Then, when MVBs are formed, they are transported to the cell membrane. The transport process involves a number of signaling proteins such as clathrin, GTPases, soluble *N*-ethylmaleimide-sensitive fusion protein attachment protein receptor (SNAREs), and coat protein complexes I and II. Subsequently, MVBs were fused or degraded to the cell membrane. The fusion process was mediated by the G protein-coupled receptor (GPCR) signal pathway, controlled by trap molecules, via phosphorylated 110 serine residues in t-SNARE SNAP23, and through the GPCR signal of the H 1 histamine receptor. Finally, the ILVs in MVBs are released into the extracellular environment in the form of exosomes and enter the systemic circulation 19.

#### Biogenesis of Microvesicles

Microvesicles (MVs) are 100-1,000 nm in size. In addition to size differences, there are also many differences between microvesicles and exosomes in proteomics and lipidomics. Compared to exosomes, which contain more extracellular matrix, heparin-binding, receptors, immune responses, and cell adhesion proteins, as well as lipids such as glycolipids and free fatty acids, MVs mainly contain endoplasmic reticulum, proteasome, and mitochondrial proteins, lipids including ceramide and sphingomyelin 20. Biogenesis of MVs begins with the plasma membrane budding, followed by vesicle release from the cell surface. Some studies suggest that MVs originate from lipid rafts rich in cholesterol and sphingolipids on the cell membrane. There is a set of signaling proteins activating MVs, and the activation of some inverting enzymes causes the rearrangement of phospholipids in MVs 21.

The key mechanism for the formation of MVs is the reorganization of the cytoskeleton, which involves the breakdown of proteins associated with the plasma membrane. There are some signal pathways involved in this process, such as the calpain-dependent pathway and Caspase-3-dependent pathway. Calpain-dependent pathway stimulates calcium intracellular flow by an agonist, activates thiol protease and calpain in the cytoplasm to move to the cell membrane. Then they bind with phosphate ester on the membrane and generates calmodulin by calcium-regulated conformational change. Activated calmodulin cleaves α-actin and talin filaments, allowing cytoskeleton proteins to be separated, thus causing MVs release. In addition, Caspase-3 cleaves the *C*-linked domain of Rho-associated protein kinase 1 (ROCK-1) and activates the phosphorylated myosin light chain (MLC) of ROCK-1, resulting in myosin interaction. RhoA/Rock signaling pathway is also involved in MVs biogenesis. RhoA is a small GTPase protein in the Rho family that regulates actin tissue and actin contractility and is involved in cytoskeleton regulation. RhoA activates the Rock (RhoA kinase), stimulates the LIM kinase, then inhibits the fibroin, reorganizes the donor cell actin cytoskeleton, and finally leads to the release of MVs 22.

#### Biogenesis of Apoptotic Bodies

**Figure 2:**
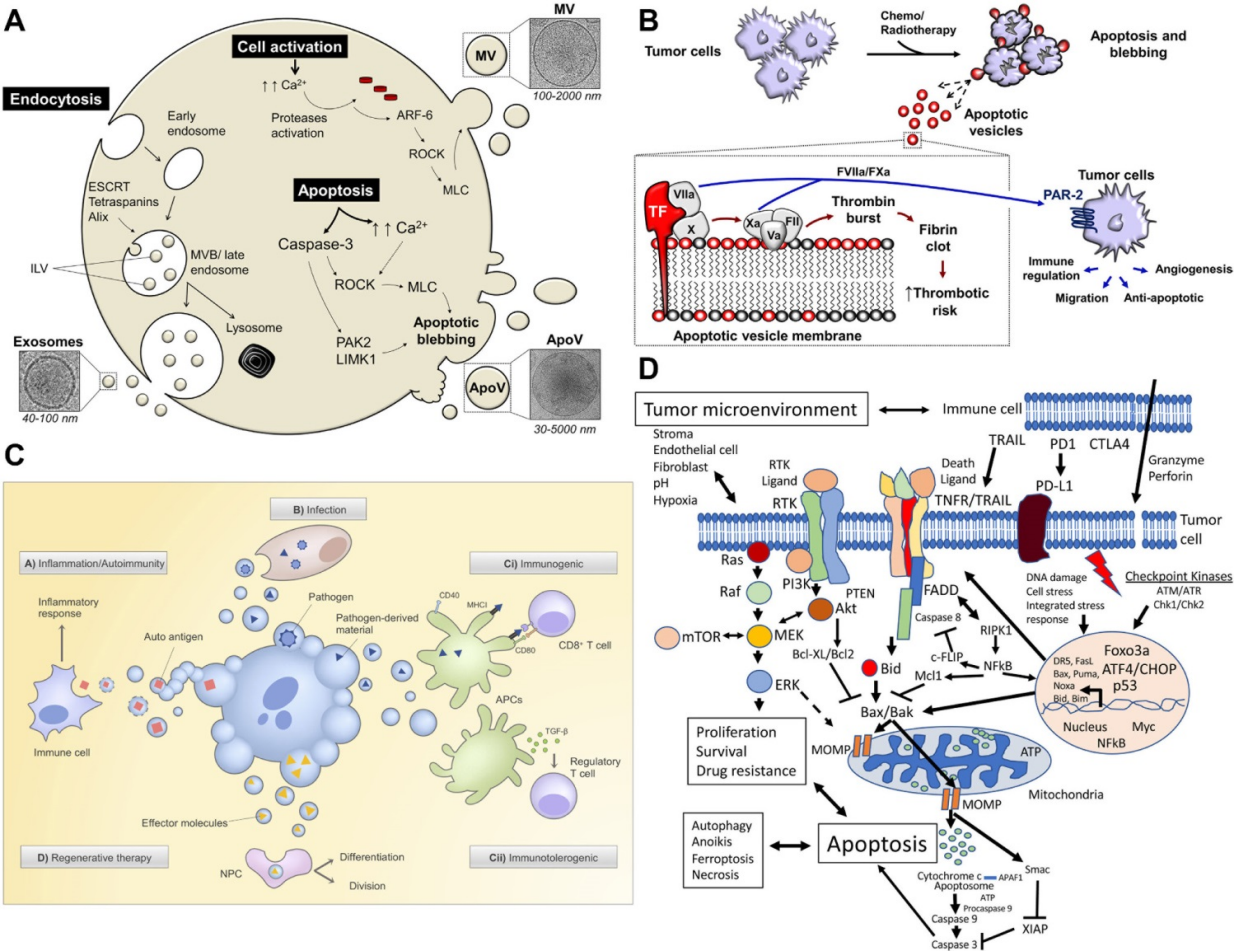
** The biological function and therapeutic potential of apoptotic bodies.** A) Apoptotic bodies are secreted by budding directly through the cell membrane. Apoptosis leads to an increase in the influx of calcium ions (Ca^2+^), which triggers proteases, and these activated proteases mediate the destruction of the cytoskeleton. ADP-ribosylation factor-6 (ARF-6) initiates the signal cascade, and finally activates the rho-related protein kinase (ROCK) signaling pathway to trigger blistering, and finally release of apoptotic bodies. The initiation of apoptosis also activates apoptotic enzymes to mediate the shedding of apoptotic bodies. B) Chemotherapy or radiotherapy-exposed tumor cells may initiate apoptosis and trigger the release ABs. Adapted with permission from 23. Copyright Year 2018, frontiers in Immunology. C) Strategies for treatment with apoptotic bodies. Adapted with permission from 24. Copyright Year 2020, PORTLAND PRESS. D) Signal pathways affecting tumor cell apoptosis. Adapted with permission from 25. Copyright Year 2020, Springer Nature.

Apoptotic bodies (ABs) are EVs produced under conditions of apoptosis. Compared with exosomes and microvesicles, apoptotic bodies are larger, with a diameter between 800-5000 nm. The main marker of apoptotic bodies is phosphatidylserine. Apoptosis is a kind of programmed cell death that does not cause inflammation. A major feature of apoptosis is that apoptotic cells eventually divide into closed apoptotic bodies. Apoptotic bodies seal and store the intracellular substances from dead cells in vesicles, and deliver these substances to other cells that can swallow apoptotic bodies, such as macrophages and tumor cells. However, the distribution of the contents of apoptotic cells to apoptotic bodies is random. Therefore, specific organelles or nuclear contents may or may not exist in a specific apoptotic body. The process of fragmentation of apoptotic cells into apoptotic bodies is conducive to the removal of apoptotic cell debris and plays a very important role in controlling immune stress after apoptosis. However, apoptotic bodies will not leak the contents of dead cells to the surrounding environment, avoiding the triggering of inflammation. Recent studies have shown that the lysis of cells into apoptotic bodies in the late stage of regulation is a highly coordinated and regulated biological process. Since apoptosis is believed to play an important role in the cell cycle and the normal development of the immune system, AB also plays a regulatory role in inflammation, autoimmune diseases, and cancer. Studies have shown that ABs derived from apoptotic bone marrow mesenchymal stem cells can enhance angiogenesis and cardiac function recovery in rats with myocardial infarction 23-25 (**Figure 2**).

### Distribution of Extracellular Vesicles

When the EVs is released by the donor cell, it begins its journey inside the body. There are many ways to label and track EVs, such as fluorescence imaging, bioluminescence imaging, nuclear imaging, tomography imaging, and so on. Zebrafish models are often used to show the distribution of EVs *in vivo*. With this high spatial and temporal resolution *in vivo* images, EVs can be observed in the manner in which they spread around the donor cell, across the biofilm, and in the organs after entering the great circulation 26.

Blood kinetic analysis showed that EVs are cleared quickly after they enter the bloodstream and have a half-life of fewer than 10 min 27. Some studies show that EVs are mainly concentrated in the spleen, liver, lung, kidney, and gastrointestinal tract, but they are most abundant in the lung. The concentration peaked at 1 h after administration and decreased after 2-12 h 28, 29. Many factors can affect the distribution of EVs *in vivo*. First, EVs from different cell sources are distributed in different locations. TDEVs accumulate more easily in tumor tissues of tumor-bearing mice, and lung metastatic hepatoma cells tend to be distributed to the lung, indicating that TDEVs have the ability to recognize early tumor tissues 30-32. Receptors on the surface of EVs may be responsible for the effect, such as increased EVs accumulation in acetylcholine receptor-rich organs after the introduction of the rabies glycoprotein target 29. Last but not least, the routes of administration and injection as well as the way the tags are tracked also have an impact on the distribution of EVs. In fluorescence imaging, CD63-based luciferase is commonly used for bioluminescence labeling. Studies have shown that NanoLuc binds to CD63 on the surface of EVs, changing the distribution of EVs, resulting in an increase in its accumulation in the lungs. It suggested that the modification of EVs tracking markers might change the distribution of organisms 33.

In particular, TDEVs can cross the blood-brain barrier (BBB). The BBB is made up of endothelial cells, pericytes, and astrocytes that are close to the brain to fight microbes and other foreign particles outside the brain. TDEVs can break through the complete BBB through endocytosis, and it can also decrease the expression of Rab7 in brain endothelial cells and improve the efficiency of transport, so as to avoid the mechanism of apoptosis of BBB hypophysis cells. TDEVs also contain proteins and nucleic acids that help to complete this process. The microRNA triggered BBB breakdown, miR-181c mediated BBB disruption by down regulating the target gene PDPK1, which regulates the abnormal restriction of actin. The miR-181c reduction of PDPK1 results in the down-regulation of phosphorylated fibroin, which in turn leads to the production of a driven fibroin that initiates the regulation of actin elements 34-36.

### Uptake of Extracellular Vesicles

After EVs are distributed *in vivo*, they are finally taken up by target cells. The uptake process consists of three stages: first, EVs target the receptor, and then, they are internalized by the recipient cells, and finally, EV content is transferred to the recipient cells. EVs can be internalized by recipient cells in three ways: endocytosis, receptor-mediated cell signaling, and phagocytosis 37. The mechanisms of EVs uptake involve a variety of membrane proteins. In the process of targeting EVs to recipient cells, the affinity between EVs membrane protein and recipient cell membrane leads to the selectivity of targeting 38. Many proteins located on the surface of EVs and recipient cells, including integrin, lectin/proteoglycan, and T-cell immunoglobulin and mucin-containing protein 4 (Tim4), are thought to be involved in the uptake of EVs. The internalization of EVs by recipient cells includes the endocytosis of both grid-protein-dependent and grid-protein-independent pathways 39. When EVs bind to the surface proteins of the recipient cells, a series of downstream signal transduction pathways are activated. It enables EVs to fuse with the plasma membrane and release the contents directly into the cytoplasm membrane. In the microenvironment of cancerous tissues, the membrane fusion in response to acidic pH is considered to be a possible mechanism of content transfer 39, 40. At present, the mechanism of EVs uptake is still under further study, and there is no systematic study to explore whether the uptake of EVs by tumor cells is different from that of other normal cells.

## Effect of Tumor-Derived Extracellular Vesicles on Tumor

**Figure 3:**
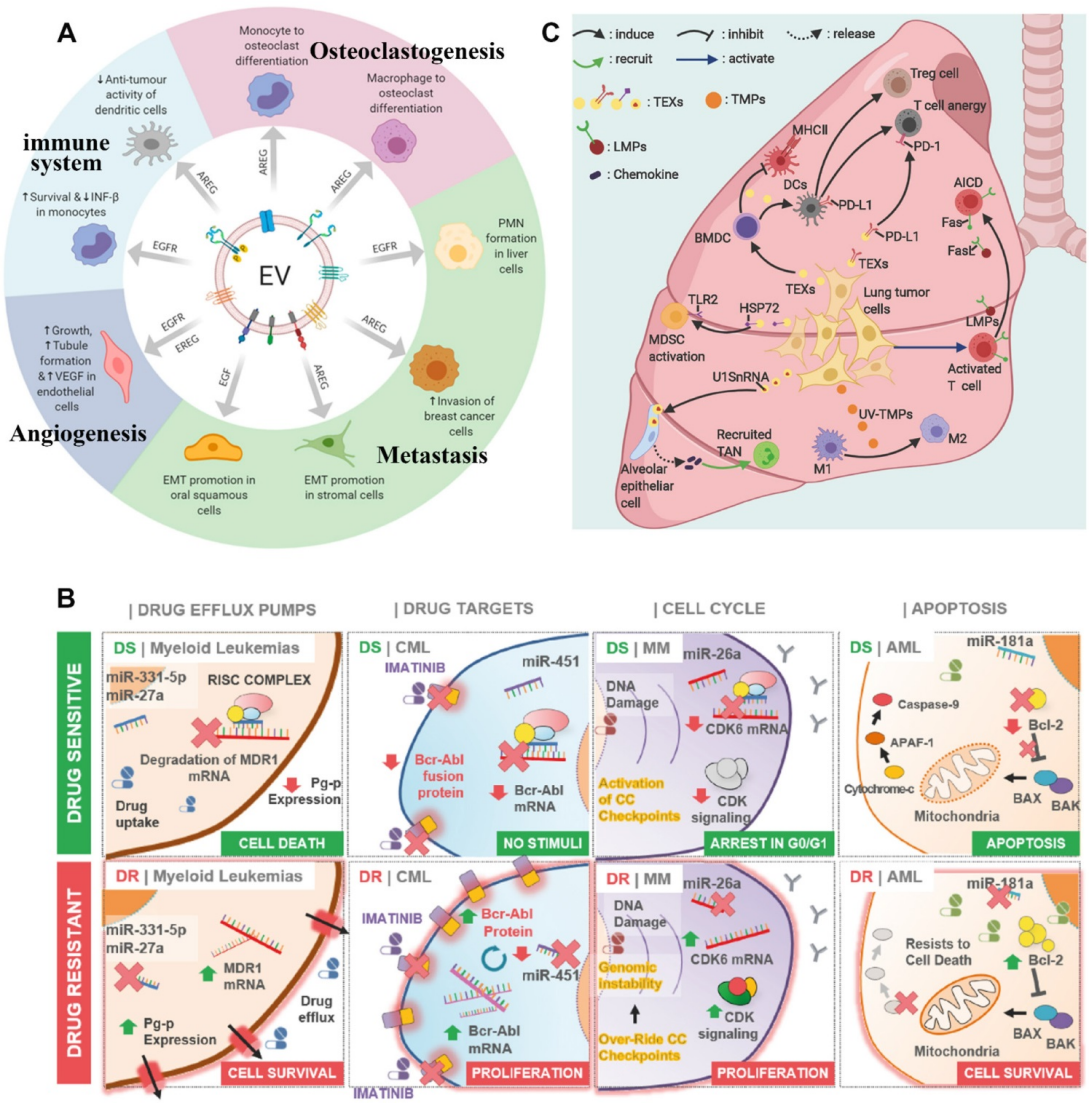
** Effect of tumor-derived extracellular vesicles on the tumor.** A) In the local microenvironment of the tumor, TDEVs regulate the metabolic state of recipient cells and promote tumor proliferation, angiogenesis, drug resistance, and immunosuppression. The role of TDEVs in the remote microenvironment is mainly to promote tumor invasion and metastasis. Adapted with permission from 62. Copyright Year 2020, MDPI. B) Mechanisms of miRNAs-mediated drug resistance in hematological malignancies. Adapted with permission from 53. Copyright Year 2021, Elsevier. C) Exosomes and microparticles derived from lung tumors can inhibit anti-tumor immunity in a variety of ways. Activated T cells release microparticles and induce their own death by FAS/FASL signal. Adapted with permission from 57. Copyright Year 2020, frontiers.

EVs are released from the donor cells and are absorbed by the recipient cells through circulation in the body, which transports bioactive substances such as proteins, lipids, nucleic acids, and metabolites from one cell to another 41. It realizes the exchange of biomolecules between the tissues of two different parts of the body. Therefore, EVs can participate in cell communication and maintain homeostasis *in vivo*. Among them, TDEVs plays the role of communication between tumor cells and other cells. Additionally, they are the vital middle person of cell-to-cell correspondence between tumor cells and stromal cells in the nearby or far-off microenvironment 42. The tumor microenvironment (TME) is a profoundly complicated heterogeneous environment made out of cancer cells, fibroblasts, adipocytes, endothelial cells, mesenchymal stem cells, and extracellular matrix. The metabolic remodeling of stromal cells is affected by cancer cells and goes about as a criticism circle to advance the development of cancer cells. Stromal cells drive metabolic changes in cancer cells and give the metabolic assets expected to cancer progression 43. Therefore, TDEVs plays a key role in the development of tumor cells. In the local microenvironment of the tumor, TDEVs can promote the growth of the primary tumor. By transferring bioactive substances, TDEVs can regulate the metabolic state of recipient cells and promote tumor proliferation, angiogenesis, drug resistance, and immunosuppression. The role of TDEVs in the remote microenvironment is mainly to promote tumor invasion and metastasis 44 (**Figure 3A**).

### Promote Tumor Growth

TDEVs promote tumor proliferation and growth. TDEVs can activate the cell pathway and induce the proliferation of tumor cells. For example, the surface of EVs derived from melanoma cells carries hyaluronic acid synthase HAS3, which contains a large number of Indian hedgehog homologs (IHH). It can activate the hedgehog signal cascade of target cells, induces c-Myc activation, and regulates the expression of cyclin. This signal transduction of IHH in HAS3-EVs leads to tumor cell proliferation and epithelial-mesenchymal transition. In addition, cancer-associated fibroblasts (CAFs) can promote the secretion of inflammatory factors and growth factors, and then promote the growth of the tumor. TDEVs are key mediators regulating cellular communication between CAFs and cancer cells. TDEVs can convert normal fibroblasts into CAFs, and cause fibroblasts to differentiate into myofibroblasts, releasing matrix metalloproteinases (MMP) and causing extracellular matrix (ECM) remodeling. ECM decomposes, causes the growth factor release, promotes the invasion ability of parenchymal cells, and promotes the tumor cell adhesion 45. For example, small extracellular vesicles (sEV) secreted by rectal cancer cells can activate human fibroblasts to differentiate into CAFs and influence the microenvironment of rectal cancer cells by reprogramming CAFs to promote tumor growth 46. In addition, the ovarian cancer (OC) cell line secretes EVs carrying miR-630 OC into nuclear factor, which promotes the activation of CAFs through the KLF6/NF-κB axis and enhances the invasion and metastasis of OC. These cases show that tumor cells can secrete TDEVs, which directly or indirectly mediates information exchange between tumor cells and their local microenvironment through different pathways, thus promoting tumor proliferation and growth 47.

### Promote Angiogenesis

TDEVs promote tumor angiogenesis. When new capillaries are formed in the tumor environment, tumor cells can enter the bloodstream and get more nutrients, oxygen, and growth factors, so new advances in cancer occur. The process of angiogenesis includes the stimulation of angiogenic factors to endothelial cells, the degradation of the vascular basement membrane, the proliferation, germination, migration of endothelial cells, the formation of the lumen, and the maturation of blood vessels. A variety of growth factors and signal pathways are involved in regulating this process. Therefore, TDEVs are indirectly involved in the regulation of angiogenesis by influencing these factors and pathways. For example, TDEVs can control the angiogenesis process by altering the net balance between angiogenic and antiangiogenic factors at the tumor site. Many kinds of nucleic acids and proteins in TDEVs can affect angiogenesis through many pathways, such as miR210, miR9, miR135b, and long non-coding RNAs such as LincPou3F3, LincRNAH19, LincCCAT2, which are transferred to endothelial cells (ECs) and activate the angiogenesis signal pathway 48. For example, when the EVs derived from glioma cells are ingested by microglia, the carried miR-21 that modulates specific downstream mRNA targets reprograms the microglia, promotes angiogenesis, and provides a favorable microenvironment for cancer progression 49. The miR-619-5p, an exocrine derived from non-small-cell lung carcinoma (NSCLC), targets RCAN1.4 and promotes angiogenesis of human umbilical vein ECs, proliferation, and metastasis of NSCLC. In addition, mRNA from TDEVs can be translated into proteins such as Wnt4 and CA9 for angiogenesis in ECs. TDEVs also stimulate angiogenesis through surface-borne angiogenic proteins such as Dll4, EGFRvⅢ, and transport anchor protein A2. TDEVs also promote angiogenesis and the production of inflammatory cells by activating the tumor-associated macrophage to secrete G-CSF, VEGF, IL-6, and TNF-α. The process of angiogenesis is complex and affected by many factors, so TDEVs can affect tumor angiogenesis through multiple pathways 50.

### Promote Drug Resistance

TDEVs promote drug resistance in tumors. The mechanisms of drug resistance include drug efflux, changes in drug metabolism, and changes in energy programming, DNA damage repair, cancer stem cells, and epigenetics 51. When TDEVs secreted by already drug-resistant tumor cells are ingested by drug-sensitive tumor cells, the nucleic acids or protein cargo they contain can alter a cell's sensitivity to a drug, making the tumor more resistant. At the same time, the receptor on TDEVs surface may antagonize the drug and reduce the concentration of the drug on the tumor cells, thus affecting the therapeutic effect 52. The miRNAs play an important role in promoting drug resistance in hematological malignancies (**Figure 3B**). Drug resistance is often associated with ATP binding cassette (ABC) transporter proteins family. Some studies have shown that miRNAs regulate the expression of some drug transporters in the ABC family after transcription, including P-glycoprotein (P-gp or MDR1), multidrug resistance-associated protein (MRP), and breast cancer resistant protein (BCRP) 53. Some miRNAs can directly target drug-efflux mRNAs. For example, TDEVs secreted by U937 cells promote efflux of the drug PEGylated liposomal doxorubicin (PLD), thereby increasing its resistance to the toxic effects of PLD cells 54. TDEVs promotes tumor drug resistance through a variety of mechanisms, which limits the treatment of cancer 55.

### Immunosuppressive

TDEVs can affect immune system homeostasis. TDEVs transport nucleic acids, lipids, and proteins mediate immune regulation in the microenvironment, and protect tumors through immunosuppression. For example, TDEVs can activate and increase Treg and myeloid-derived suppressor cells (MDSCs) and inhibit CD8^+^ T cell-mediated tumor-targeting immunity. The apoptosis of CD8^+^ T cells was directly induced by the expression of FasL and TNF-related apoptosis-inducing ligand (TRAIL). TDEVs increases neutrophil promotes tumor progression and activates or suppresses natural killer (NK) cells, which play an important role in tumor immunity. The antigen delivered by TDEVs can activate dendritic cells (DC) and participate in CD8^+^-mediated anti-tumor response 56. Many studies have shown that TDEVs from lung tumor cells or immune cells promote tumor progression by inhibiting anti-tumor immunity (**Figure 3C**). During immune editing, TDEVs may act as an immunostimulator for cancer cells to germinate and then transform into an immunosuppressive factor during the progression of cancer. In the clinical application of cancer vaccines based on TDEVs, it is necessary to pay attention to its function of inhibiting tumor immunity 57.

### Promote Tumor Metastasis

In the tumor distant microenvironment, TDEVs mainly showed the promotion of pre-metastatic niche (PMN) for tumor formation. In the process of tumor metastasis, TDEVs released by tumor cells *in situ* can induce vascular leakage and interact with resident cells of distal organs to promote the proliferation of TDEVs *in vivo* 58. TDEVs membrane contains a series of integrins, which can target specific types of resident cells in specific organs and mediate tumor metastasis to specific sites, such as avb5 mediates liver metastasis, α6b4, and α6b1 mediate lung metastasis 59. Lipid rafts on the TDEVs membrane may also be involved in the activation of tumor metastasis signaling pathways and are regulated by ceramide. Then, when TDEVs is ingested, it can induce the inflammatory factors S100, TGF-b, IL-6, IL-8, TNF-α, and so on. These factors can lead to the remodeling of stromal cells in the distant microenvironment, forming the pre-metastasis niche and constructing a favorable ecological environment for the growth of new tumors 60. For example, exosomes derived from gastric cancer cells remodel the tumor microenvironment by inducing autophagy and pretumoral activation of neutrophils through the HMGB1/TLR4/NF-κB signal 61. The epidermal growth factor receptor (EGFR) is an important agent in this process. TDEVs transmit EGFR or EGFR ligands to promote metastasis, premetastatic niche formation, osteoclast formation, angiogenesis, and immune regulation 62. In addition, a viral oncogene latent membrane protein 1 (LMP1) was modified on the surface of EVs, resulting in changes in the ESCRT mechanism secreted by these EVs, which promoted cell adhesion, proliferation, migration, and tumor growth 63.

Notably, TDEVs tend to be ingested by tumor cells *in vivo*, and compared with other tumors, TDEVs are more likely to be ingested by the same type of tumor cells. In zebrafish embryo models, TDEVs were absorbed by endothelial cells and macrophages, stored in degradation chambers, activated macrophages, and ultimately promoted tumor metastasis 64, 65. The specificity of uptake may be related to a number of factors, and studies have shown that highly selective nanoparticles are present in the blood of cancer patients to transport TDEVs 66. Abnormal glycosylation of tumors may also play a role 67. For example, EVs secreted by B16F10 melanoma cells are preferentially ingested by autologous melanoma cells, but not by colon cells, macrophages, or renal cell sarcomas. This tumor uptake specificity enables information to be exchanged between tumors in different parts of the body 66.

In summary, TDEVs play an important role in the progression of cancer. Tumor cells can both directly or indirectly affect tumor cells and tumor microenvironment through TDEVs-mediated signaling pathways. Therefore, the TDEVs-mediated tumor cell-to-cell pathway is not only a target for cancer therapy in the strategy of tumor therapy. At the same time, when TDEVs is used as a drug carrier to treat cancer, the TDEVs cargo can be changed or surface modified, so that the therapeutic effect of TDEVs drug carrier system cannot be counteracted by its promotion of tumor development 68-70.

## Construction of Tumor-Derived Extracellular Vesicles as Drug Delivery System

### Isolation of Extracellular Vesicles

**Figure 4:**
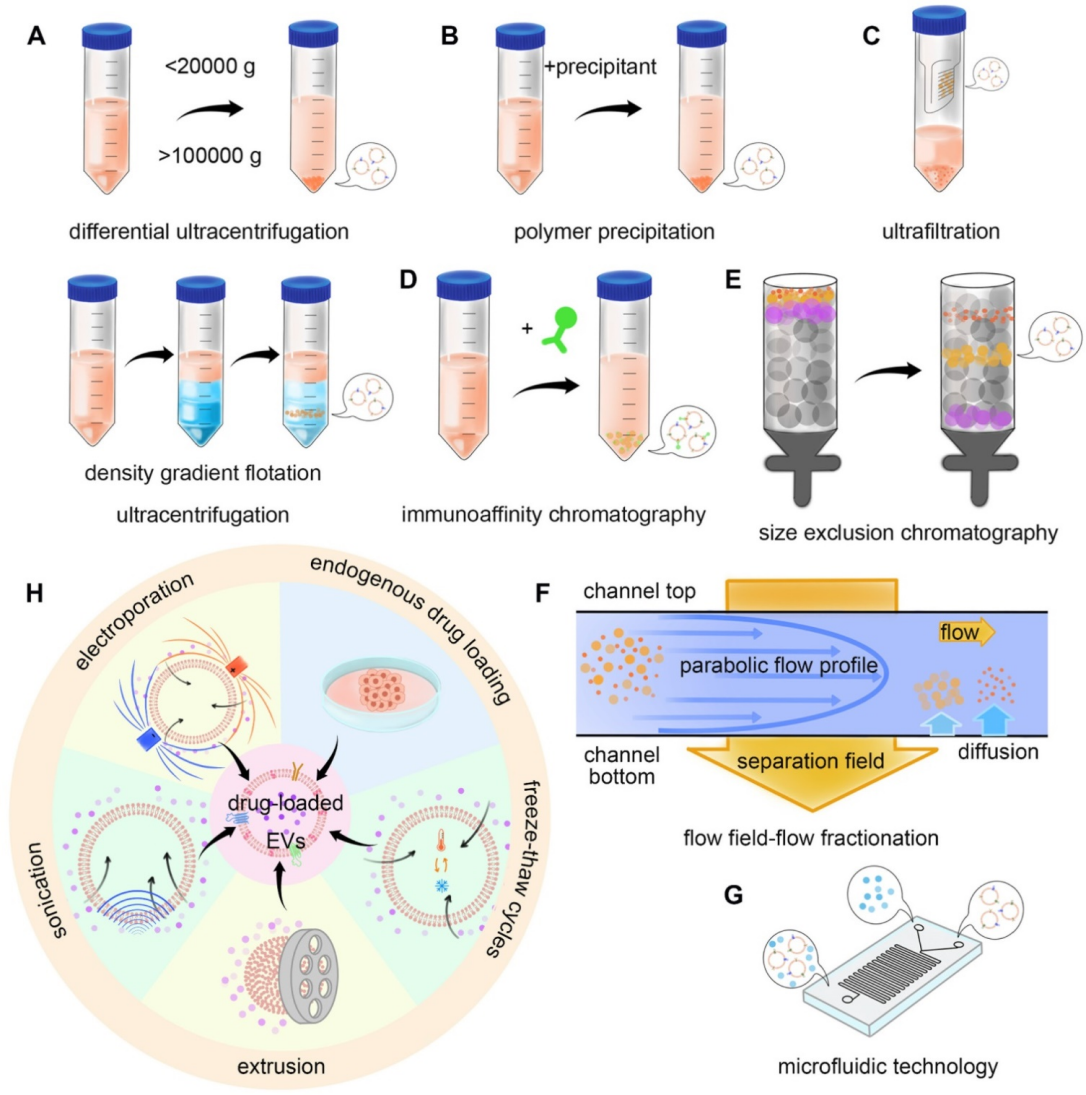
** Isolation and drug loading methods of EVs.** The commonly used isolation methods mainly utilize the physical properties and biological specificity of EVs. For example, A) differential ultracentrifugation (DU) and density gradient flotation (DGF), B) polymer precipitation-based isolation, C) ultrafiltration, D) immunoaffinity chromatography, E) size exclusion chromatography (SEC), F) flow field-flow fractionation (FFFF), and G) microfluidic technology can realize the isolation of EVs. H) Drug loading methods can be divided into endogenous and exogenous. Endogenous drug loading enables the drug to be added into the source cells of EVs, while exogenous drug loading directly loads the drug into EVs. Specific methods include endogenous drug loading, incubation, electroporation, sonication, extrusion, and freeze-thaw cycles.

The construction of the TDEVs as DDS starts from the isolation and extraction of EVs. Several methods can be used for the purification of EVs. The usual methods mainly utilize the physical and biological characteristics of EVs. Based on the physical characteristics of separation methods include the use of EVs density or size and impurities for separation, such as ultracentrifugation, size exclusion chromatography, ultrafiltration, and flow field-flow fractionation. The method based on biological characteristic separation mainly utilizes the specific molecules on the surface of EVs, including polymer precipitation and the immunoaffinity method. There are also integrated approaches such as microfluidic technology 71. In 2019, the results of a global survey on techniques for the isolation and identification of EVs conducted by the international association of extracellular vesicles showed that ultracentrifugation and density gradient centrifugation were still the most common methods, with the increasing use of size exclusion chromatography, microfluidic technology is attracting more and more attention 72 (**Figure 4A-G**).

#### Ultracentrifugation

Ultracentrifugation is a kind of separation method based on the difference in particle density and size. There are two types of ultracentrifugation: differential ultracentrifugation (DU) and density gradient flotation (DGF). Ultracentrifugation is the most common technique for the separation of EVs from cell culture supernatants or other biological fluids. However, it has the disadvantages of time-consuming operation, limited production, low automation, and dependence on expensive instruments.

In DU, the usual procedure for separation is the removal of large cell fragments and cells at a low speed (less than 20,000 × g), followed by further removal of proteins by precipitation of EVs at a high speed (more than 100,000 × g). However, the disadvantage of DU is the operation time is long, which may damage the integrity of EVs, resulting in protein contamination and yield reduction 73. DU is suitable for the extraction of EVs with less plasma protein and lipoprotein contamination. After separation, a washing step can be added for further purification. The yield and purity can also be improved by one-step sucrose buffering before centrifugation 74.

DGF is another kind of ultracentrifugation. The first step is to construct a density gradient. Common solvents include sucrose and iohexanol, both of which are viscous solutions, with the latter having a lower viscosity. In the solution with an increasing concentration gradient, EVs can migrate to their equilibrium density after centrifugation, and the migration velocity depends on its size, shape, and density. By using this technique, the protein contamination in the sample can be eliminated and therefore, be more effective than DU. One study compared the difference between DU and DGF with Optiprep^™^ for isolating EVs secreted by leech microglia, the results showed that DGF was superior in removing protein impurities and other membrane particles 75.

#### Size Exclusion Chromatography

Size exclusion chromatography (SEC) is a method of separation using particle size and shape. In this method, small porous polymer beads were used as stationary phphaseor separation of EVs 76. When solutions containing particles of different sizes and shapes pass through the stationary phase, smaller particles can enter the small porous polymer beads, while larger or irregularly shaped particles cannot, but can only follow the mobile phase through the separation column. Therefore, the larger particles pass through the column faster than the smaller particles and are washed out earlier 77. SEC is widely used in the separation of EVs. This method has the least damage to EVs and can keep EVs bioactivity. SEC removes most of the soluble protein impurities that do not bind to EVs, especially plasma protein contamination, increasing the likelihood of expanded EVs recovery at high-quality standards 78.

Compared with the DGF method, the SEC method is less time-consuming, more convenient, and more automatic 79. After analyzing the microRNA and protein spectra, we found that the separation efficiency of SEC was equivalent to that of gradient centrifugation 80. Compared with rhesus monkeys and mice, the EVs extracted from human tissue are purer, which indicates that this method has the opportunity to be more optimized. Overall, the SEC is an approach that has a big advantage 81. A study using SEC to separate EVs from human synovial fluid found that DU did not remove large amounts of the albumin, high-density lipoprotein, apolipoprotein, fibronectin, and other extracellular proteins and fragment contaminants, can be removed by the SEC method. As a result, the SEC extracts EVs with higher purity 82. In addition, SEC can obtain more complete biophysical properties from cardiac myocytes-derived EVs. Compared with ultracentrifugation, there are 17 functional differences, so SEC is not easy to destroy EVs activity. Also, the TDEVs isolated by SEC still retained the activity of tumor recognition 83. A recent study optimizes the clinical use of SEC. The performance of three Sepharose CL resins (CL-2B, CL-4B, and CL-6B) was compared and the column bed volume was improved. The results showed that the two-step batch elution of two-minute SEC was sufficient to recover EVs with high purity and particle recovery from fetal bovine serum (FBS), human serum (HS), and FBS-free cell culture supernatant 84.

#### Ultrafiltration

Ultrafiltration is also a method of separation based on particle size. The principle of separation is that the sample passes through a semi-permeable membrane with certain pore size. The particles with smaller pore sizes pass through the membrane, while the particles with larger molecular weight are retained. The extraction of EVs by ultrafiltration has the characteristics of high efficiency, not easy to destroy the structure of EVs, and high yield and stable EVs can be obtained.

The separation efficiency of EVs by ultrafiltration is similar to that of SEC. The results of electron microscopy and nano-tracking analysis showed that the number of exosomes extracted from human melanoma cells by ultrafiltration and SEC was 58 times as much as that by ultracentrifugation. It shows the efficiency advantage of EV extraction by ultrafiltration 85. In addition, some automatic separation systems, such as exosome total separation chip (ExoTIC), can be designed according to ultrafiltration. A nanoporous membrane was installed to enrich EVs in the range of 30-200 nm. The chip has the advantages of simple use, high yield, and high purity 86. Exodisc-b is also an automated device for separating whole blood EVs. The device has a nanoporous filter with a minimum sample size of 30 μL and an efficiency of over 75% for EVs separation 87.

#### Flow Field-Flow Fractionation

Flow field-flow fractionation (FFFF) is also a method of separation based on particle size. In this method, particles of different sizes are loaded into a small channel made up of two semi-permeable membranes. The liquid then flows because it interacts differently with particles of different sizes, and particle mobility is also affected by the size, which can distinguish different particles. Asymmetric flow field-flow fractionation (AF4) is an advanced FFFF technology. AF4 has high reproducibility and purity in the process of removing high-density lipoprotein and low-density lipoprotein from plasma to obtain EVs 88. Optimization of cross-flow gradient, focusing time, ultrafiltration conditions, sample size, and injection volume can continue to improve the separation efficiency 89.

The method of separating EVs based on physical properties is to purify EVs by taking advantage of the difference between EVs and impurities in size, shape, or density. The advantages of the physical separation of EVs are high yield and efficiency, and the structure of EVs is not easy to be destroyed 90. However, in some cases, some protein contaminants overlap with the size distribution of EVs, resulting in a decrease in the purity of EV products. Therefore, how to improve the separation effect of EVs effectively is still a challenge. At present, the effective solution is a combination of multi-step and multi-model. For example, the purity of EVs extracted from urine by ultrafiltration and SEC combined with AF4 is much higher than that obtained by a single method 91. Continuous centrifugation and ultrafiltration were used to isolate the EVs secreted by human glioblastoma multiforme stem cells and to identify the microvesicles and exosomes subtypes 92. Size-based EVs purification can also be designed as an automated operating system, such as Exo-pos. The system can effectively separate EVs with high purity and integrity as a specific biomarker from complex biological fluids 93.

#### Polymer Precipitation

Polymer precipitation separation is a method of separation by using the biological properties of EVs. In this method, a special polymer is dissolved in the liquid, which reduces the solubility of the EVs and precipitates out. For example, polyethylene glycol (PEG) can precipitate EVs. Low-speed centrifugation can accelerate the separation of EVs when using PEG to precipitate EVs 94. However, this method has some disadvantages. Some protein impurities can also be precipitated by PEG, resulting in a decrease in the purity of EV products. Therefore, the purity of PEG can improve by subsequent washing after precipitation. One study collected EVs from cell culture supernatants and found that PEG products contained a percentage of non-EVs-related molecules. The protein impurities in EVs were reduced more after ultracentrifugation. For example, the combination of the polymer precipitation method and SEC method can significantly improve the separation efficiency of EVs 95.

There are some commercial EVs extraction kits, such as Exo-Quick, ExoSpin, and so on, which use the principle of polymer precipitation to extract EVs 96. There are also kits such as qEV35 and qEV70 that take advantage of the SEC principle of EV extraction. The EV protein extracted by the SEC kit had less pollution and higher purity, and the yield of qEV35 was higher than qEV70 97. In the experiment, we should choose the right reagent box according to the actual situation. However, polymer precipitation changes the state of EVs, which may affect the original structure of EVs and reduce their stability.

#### Immunoaffinity Chromatography

Immunoaffinity chromatography is a separation method based on the specific affinity of EV surface molecules. Some membrane surface proteins or lipids, such as tetraspanins, glycosylphosphatidy-linositol-anchored protein (GPI-Aps), and tumor necrosis factor receptor 1 (TNF-R1), are known as specific binding sites. There are some extraction kits based on the immunoaffinity method, such as PAP/ME kits. Compared with the methods of ultracentrifugation and SEC, the yield of EVs obtained by the immunoaffinity method is lower, but the protein contamination is less 98.

Because immunoaffinity assay is a method of using special proteins on EVs, different EVs membranes from different sources may contain different proteins. Therefore, on the one hand, immunoaffinity may not be able to extract all the EVs, resulting in reduced extraction efficiency. On the other hand, this property can be used to extract specific sources of EVs. For example, monoclonal antibody 763.74 against a uniquely expressed chondroitin sulfate proteoglycan 4 (CSPG4) epitope on melanoma cells. Using immunoaffinity, the exosomes derived from melanoma cells were isolated from the plasma of patients with melanoma, and the exosomes from other sources were removed 99. Plasma immunoaffinity assay can also be combined with other methods to improve the effect. Combined with the SEC and immunoaffinity methods, impurities such as virus particles, viral hepatitis type B (HBV) surface antigens, HBV core antigens, antibodies, or infectious substances can be removed from hepatitis B virus plasma-derived EVs. The method has high efficiency, good repeatability, and stable result 100. One immunoaffinity chromatography method has been developed for the separation of plasma-derived EVs containing CD61 by immobilizing CD61 antibodies onto the eisk monolithic column. The whole process takes only 19 min, and the efficiency can be further improved by increasing the flow rate 101.

#### Microfluidic Technology

In recent years, separation methods based on microfluidic technology have been paid more and more attention. Microfluidic technology is to uses a continuous flow process to distinguish different sizes of nanoparticles, and then according to the surface markers, ligands, charges, particle size, and so on to identify the required EVs. The method has the advantages of the short operation time, less consumption of reagent, and sample volume 102-105.

Microfluidic technology can be combined with the immunoaffinity method to design a variety of microfluidic devices. This kind of device can be combined with a diverse of physical or chemistry principles 106, 107. In one study, cancer-secreting EVs expressed phosphatidylserine in the lipid bilayer specifically. Based on this, an immunoaffinity microfluidic system, ^new^ExoChip, was developed to isolate cancer-related exosomes from plasma. The capture efficiency of TDEVs and healthy exosomes was 90% and 38% respectively 108. Melanoma-specific antibodies MCAM and MCSP in the device can isolate circulating tumor cells (CTC) and exosomes in the OncoBean (DUO) apparatus 109. Click chemical reactions can also be used in microfluidic devices. The EVs chip (EVOD) utilizes a catalyst-free click-chemical reaction to quickly separate specific EVs. Antibodies that bind cancer-related surface proteins in a click chemical reaction. The number of EVs isolated from small-cell carcinoma cells was 76% higher than that from normal cells 110. It is important to note that after the antibody binds to the EVs to be separated, the captured EVs need to be released from the substrate. How to destroy the binding between antigen and antibody without changing the original structure of EVs is a problem. The OncoBean (DUO) device can be improved on the basis of the original by binding the antibody coupled with the disulfide biotin 111.

EVs can be separated in a number of ways. Therefore, in the preparation of DDS, the first step is to select the appropriate separation method according to the need and the actual situation. The separation method based on EVs' physical properties has the advantages of high yield, stable properties, but low purity. The separation method based on the biological characteristics of EVs has high purity and specificity, but the yield may be damaged 112, 113. In addition, the type of biological fluid sample, the type of impurity, and the subsequent requirements need to be considered when selecting the appropriate separation method 114.

### Drug Loading Method

After extracting enough EVs to meet the requirements, the drug can be loaded. Drug loading methods can be divided into endogenous drug loading and exogenous drug loading. The exogenous drug loading methods include incubation, electroporation, sonication, extrusion, and freeze-thaw 115-117 (**Figure 4H**). This section introduces the related research progress (**Table 1**).

#### Endogenous Drug Loading

Endogenous drug loading means that the drug is first added to the medium and cultured with the cell. In the physiological activities of the cell, the drug is taken up automatically. As the cell secretes EVs, the drug enters EVs along with the cytoplasm, so the cell can secrete drug-loaded EVs. The successful preparation of drug-loaded EVs involves the successful uptake of the drug by the cell and its excretion by the cell via EVs. Therefore, if choose the endogenous drug-loading method to prepare DDS, the first step is to prove that the drug can be successfully transported through the biogenetic pathway of EVs. The release of drug-loaded EVs may involve autophagy. Autophagy is a highly regulated homeostatic process that prevents cell damage by removing, degrading, or removing damaged cell components. The mechanism involves the biogenesis of EVs. During the formation of ESCRT, there is a protein Ndfip1 with L-domain, which helps drugs enter EVs 118.

#### Incubation

In addition to the endogenous drug loading method, an exogenous drug loading method can also be used. The EVs were first isolated from the cells and then loaded drug by a series of drug-loading methods. The commonly used drug loading methods include incubation, electroporation, sonication, extrusion, and freeze-thaw. Different types of drugs should be loaded in different ways. For example, some hydrophobic or small-molecule drugs can enter EVs passively, so they can be loaded by simple incubation. On the contrary, hydrophilic compounds cannot passively pass through lipid vesicles. So a proactive approach is needed to enable drugs to penetrate the pores of the EVs membrane. The principles of electroporation, sonication, extrusion, and freeze-thaw are to create pores on the surface of EVs with sufficient pore size to allow drugs to enter 119.

Some hydrophobic molecules, such as paclitaxel (PTX), doxorubicin (DOX), and curcumin, can be passively loaded into EVs by co-incubation with EVs. EVs were first isolated from the culture supernatant of Raw264.7 cells in the construction of EVs vector for the treatment of methicillin-resistant staphylococcus aureus infection. After the incubation with linezolid antibiotics at 37ºC for 1 h, the unbound drugs were removed by 10,000 × g low-speed centrifugation for 10 min and then sterilized by 0.22 μm microporous membranes, to get an EVs loaded with antibiotics. The drug-loaded EVs were 5.06% ± 0.45% by HPLC analysis 120. The advantage of incubation is that it does not change the structure and properties of the drug and EVs, but its application is limited to small hydrophobic molecules that can be loaded passively. In order to improve the loading efficiency, EVs can be protonated to produce a pH gradient on the membrane, which can enhance the EVs' loading of nucleic acid drugs 121. In addition, some compounds can aid incubation, such as glycosides-assisted hydrophilic porphyrins loaded into EVs that can increase drug loading by 11 times. Polyethylene glycol-based therapeutic nanoparticles (NPs) show up to 50% internalization 122, 123.

#### Electroporation

Electroporation is an extrinsic drug loading method in which a change in voltage caused by an electrical signal leads to the formation of temporary pores in the EVs membrane to allow drugs to enter. Electroporation is often used for loading nucleic acid drugs, but it may cause nucleic acid precipitation or vesicle fusion. Therefore, in recent years, some new drug loading methods and facilities have been developed based on the principle of electroporation 124-126. As the electroporation of large messenger RNA will lead to the decrease of the yield of drug-loaded EVs, a cell nano-modification method has been developed as an improvement measure. First, all kinds of cells were transfected with plasmid DNA, which stimulated the release of transcription RNA-carrying EVs by local and transient electrical stimulation. The yield was increased by more than 50 times, and the mRNA content was increased by more than 10^3^ times 124. In addition, electroperforators are expensive, and a simple and inexpensive handheld ElectroPen has been developed for this purpose. It can provide about 2,000 V repeatable exponential attenuation pulse in 5 ms. The efficiency of the device is comparable to that of the traditional electroperforator, but the cost is reduced considerably 127.

#### Sonication

The sonication method is a kind of exogenic drug loading method, which can form pores on the membrane by vibrating EVs with periodic ultrasonic waves. In a study of DOX loading for tumor targeting, DOX-loaded EVs were prepared using a sophisticated ultrasound device with 20% amplitude and /150 sec. 6 cycles for 30 sec. The setting of sonication parameters directly affects the result of drug loading. The disadvantage of the sonication method is that it may change some physical properties of EVs, such as size, electric potential, and so on. Therefore, the optimization method based on traditional ultrasound improves the drug loading effect. One study combined microfluidics and sonication to prepare poly (lactic-co-glycolic acid) (PLGA) NPs coated with the exosomal membrane (EM) or cancer cell membrane (CCM) to reduce drug clearance and improve tumor-specific targeting 128.

#### Extrusion and Freeze-Thaw

Other methods include extrusion and freeze-thaw, but these two methods are not often used for TDEVs drug loading. According to the nature of drugs, different methods should be chosen for drug loading 129. A study evaluated four methods of adding catalase to exosomes: co-incubation with saponin, freezing and thawing, ultrasound, and extrusion. The results showed that the loading efficiency of exo-CAT obtained by ultrasonic, extrusion, and saponin treatment was higher, and the catalase activity was not affected. However, the effect of freeze-thaw treatment was bad 130. Another attempt was made to encapsulate HGNs (which absorb light from the NIR region for selective thermal ablation) in a mouse melanoma cell-derived exosome (B16-F10-exos). The experimental methods include electroporation, endogenous drug loading, thermal shock, ultrasound, and saponin-assisted drug loading. The results showed that although almost all methods could be used to load the drug, they had adverse effects on the morphology and integrity of the exosomes 123.

### Engineering Methods

Drug-loaded EVs can be modified on the surface of the membrane, which is helpful to improve the targeting, change the biological distribution and improve the curative effect 141. Common surface modification methods can be classified according to technical principles, such as endogenous engineering, or directly acting on the modification of EVs, which includes physical modification, chemical modification, and membrane fusion technology 142.

#### Endogenous Engineering

**Figure 5:**
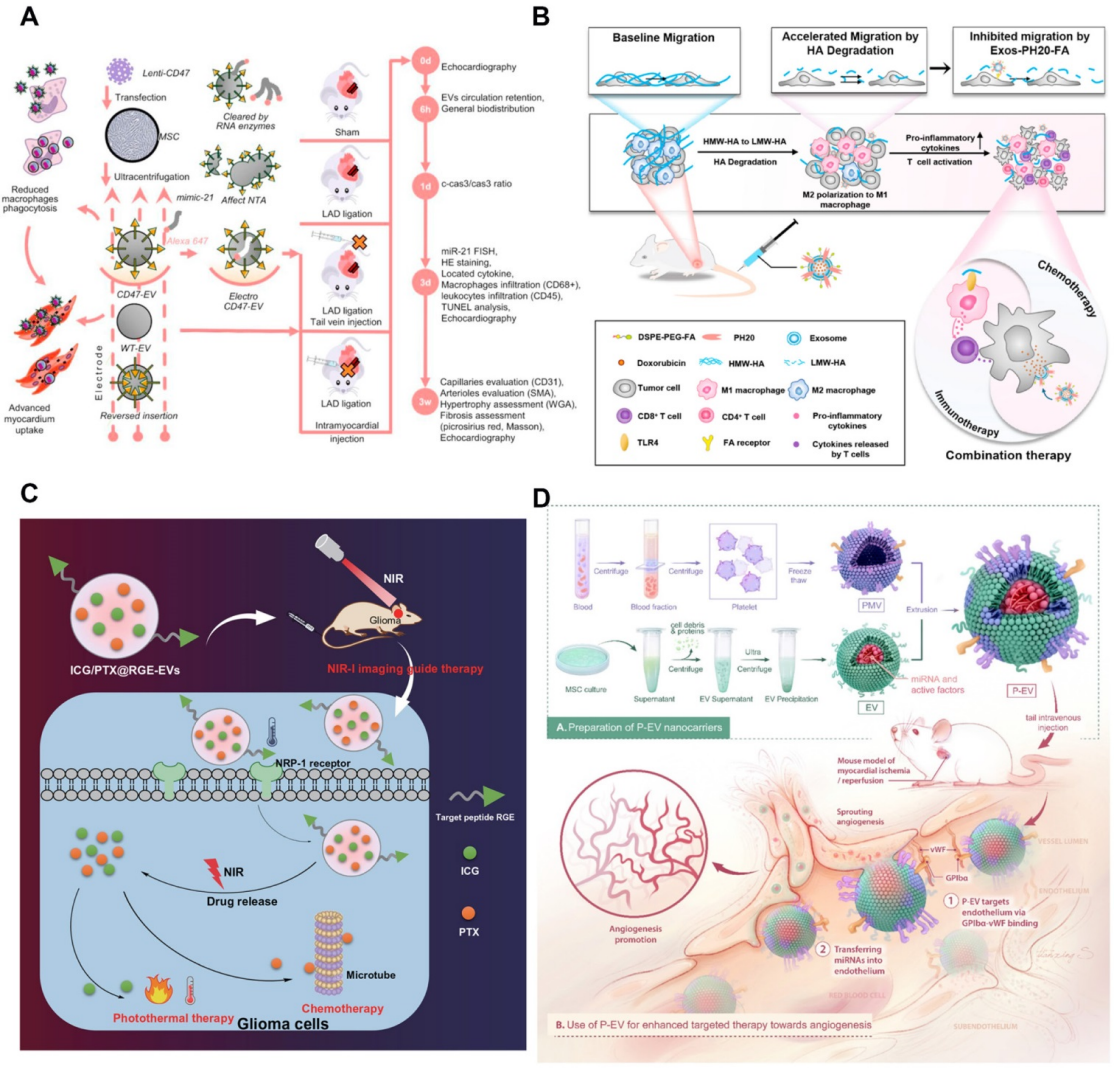
** Engineering methods of tumor-derived extracellular vesicles.** A) Schematic illustration of the EVs modified with CD47 on membrane surface to achieve mononuclear phagocyte system blockade. Adapted with permission from 147. Copyright Year 2021, Elsevier. B) The effects of DOX@Exos-PH20-FA on TME regulation programs indicate that this leads to increased uptake of DDS by tumors and a shift in the immune microenvironment from immunosuppression to immune support for cancer treatment. Adapted with permission from 152. Copyright Year 2021, Elsevier. C) ICG/PTX@RGE-EV can effectively target glioma to enact the Caspase-3 pathway through chemotherapy-hyperthermia in commitment to glioma cell apoptosis, which implies cancer development is smothered. Adapted with permission from 161. Copyright Year 2021, Springer Nature. D) Schematic diagram of P-EVs fabrication and its targeted therapy towards angiogenesis. Adapted with permission from 167. Copyright Year 2021, IVYSPRING.

Endogenous engineering is a kind of genetic engineering technology that can change the gene sequence of donor cells to change the structure of secreted EVs. The spatial conformation of transmembrane proteins that are selectively enriched in EVs is the same as the donor cells. As a result, some transmembrane proteins can be used to express ligands or homing peptides on the surface of EVs, such as tetraspanins (CD63, CD81, and CD9), CD protein (LA), and lysosome-related membrane protein-2b (Lamp-2b) 143, 144. The endogenous engineering is mainly to insert the cDNA sequence encoding homing peptide into the sequence encoding EVs membrane protein signal and the sequence encoding the *N*-terminal of the mature peptide by gene engineering technology. Therefore, it can express the target homing peptide on the EV membrane 145. The common vector of transfection is a plasmid. For example, the donor cells were genetically engineered to exocrine rabies virus glycoprotein (RVG) peptides targeting α7-nAChR, and a highly specific variant of enkephalin (Aβ) was added to the surface of the exocrine. When the drug was administered to the whole body of the mouse, the drug-loaded exosomes first targeted the hippocampus of the brain, significantly improving the targeting ability 146. Another study overexpressed CD47 on the surface of mesenchymal stem cells by genetic modification and then extracted the EVs from the cells to get the EVs that had expressed CD47. Then load miR-21 into it and build DDS. CD47-mediated signaling pathway can evade the clearance of macrophages, effectively internalize into cardiomyocytes, and inhibit cell apoptosis 147 (**Figure 5A**).

#### Physical Modification

Physical modification is a kind of method which directly affects the surface molecule of EVs membrane. Specifically, lipo-solubility molecules were anchored into the bilayer membrane of EVs, and the targeting of EVs was improved by rearrangement of the bilayer membrane 148, 149. Cholesterol and synthetic phospholipids are the most commonly used lipo-solubility crosslinkers. For example, a targeted ligand can be added to EVs membrane by binding to a compound that can be inserted into the membrane. Nano-sized PEG micelles were prepared by binding epithelial growth factor receptor (EGFR) with phosphatide (DMPE). After the micelles were mixed with the EVs from Neuro2A cells or platelets, the transfer of the nano-PEG-lipid to the EVs membrane was observed to be temperature-dependent. The specific binding of the modified EVs to EGFR overexpressed tumor cells was significantly increased 150. The incubation process can help to realize the physical modification. Co-incubated the lipoid-chain grafted HA (lipHA) with non-cancerous HEK293T cells secreted hEVs, and modify hyaluronic acid (HA) onto EVs to produce lipHA-hEVs. It can effectively transport the DDS to the tumor site. The intracellular DOX accumulation in multidrug-resistant breast cancer cells was promoted significantly 151. Similarly, Exos-PH20-FA was prepared by using the plasmid transfection method and self-assembly method respectively with human hyaluronidase (PH20) and folate (FA) on EVs membrane to inhibit the metastasis of tumor cells induced by hyaluronidase. It enhanced the delivery of chemotherapy through FA-modified tumor targeting 152 (**Figure 5B**). In addition, there are also studies on the surface of EVs to prepare a film, EVs included in the internal, forming a protective barrier. For example, the nano-film prepared by Fe^3+^ supramolecular complex and tannic acid can encapsulate exosomes, protect the exosomes from ultraviolet radiation or heat damage, and control the drug release. Gold nanoparticles are also attached to the nano-film to serve as visual markers 153. Gold nanoparticles can also grow a popcorn structure around the EVs. Nano-gold has the ability of light and heat transfer. Under near-infrared irradiation, it can produce hyperthermia, induce tumor ablation, and trigger drug release 154. The surface of EVs can also be coated with a hydrogel-coupled poly (ethylene glycol) chain (Upy hydrogel) based on the Uracil (Upy) unit, which changes the gel state from high pH to neutral pH, thus prolonging the time of EVs exposure to target organs 155.

#### Chemical Modification

Chemical modification is a method to modify the molecular structure of EVs film by chemical reaction. It is a kind of chemical reaction with high efficiency and strong anti-interference. This method is based on the synthesis of small units, with the click of a chemical reaction to obtain a diversity of molecules 156-158. Copper-free click chemistry, for example, couples EVs containing azide lipids with targeted peptides to improve the targeting of cancer cells 159. The preparation of membrane-targeted cytopenetrating peptide (CPP) and TNF-α anchored exosome binding superparamagnetic iron oxide nanoparticles (CTNF-α-exosome-spins) via transferrin-transferrin receptor (Tf-TfR) interaction. The transferrin-modified SPIONs (Tf-SPIONs) were combined with CTNF-α-exosomes to prepare drug-loaded exosomes, which significantly enhanced the growth inhibition of tumor cells 160. In another study, EVs containing indocyanine green (ICG) and PTX were first prepared, and then Neuropilin-1 targeting peptide (RGE) was coupled to ICG/PTX@EV via a cyclic addition reaction of sulfonyl azides according to the click chemical method. ICG/PTX@RGE-EV shows good photothermal properties and drug release ability. Furthermore, this DDS can target U251 cells completely, activate the Caspase-3 pathway through chemotherapy and hyperthermia, and enhance U251 cell apoptosis 161 (**Figure 5C**).

In addition to the addition of specific ligands to the surface of EVs, it is also possible to remove the endogenous substances on the surface of EVs, such as stripped surface glycans. As mentioned above, glycometabolism not only regulates the biogenesis of EVs, but also the distribution of EVs *in vivo* may be affected by surface glycosylation modification 162. Therefore, it is possible to change the targeting of EVs by changing the surface glycosylation modification. The most effective way is to remove sialic acid residue, which can increase the accumulation of EVs in the lungs 163.

#### Membrane Fusion Technology

The membrane fusion technology is to mix EVs membrane with another lipid membrane from different sources to get a hybrid membrane. The hybrid membrane has the same characteristics as the original lipid membrane. It can also be used for the combination of bio-membrane and liposome 164, 165. By fusing a simple EV-plasma membrane with EVs that overexpress HER2 in BT-474 cells, a sufficient amount of HER2 could be successfully implanted on the surface of TNBC MDA-MB-231 cells. Subsequently, anti-HER2 antibodies were coupled with PTX-loaded liposomes for HER2-targeted drug delivery 166. Membrane extrusion technology can also be EVs surface composition and lipid material fusion, mass production engineering EVs. The lipid libraries (DOTAP, POPC, DPPC, and POPG) were fused with EVs to form a mixed lipid membrane structure. For example, to enable EVs to have the target abilities of platelets, platelet-mimetic EVs (p-EVs) can be prepared by fusion of EVs with the platelet membrane using extrusion. The modified EVs have the function of targeting endothelial cells and promoting angiogenesis 167 (**Figure 5D**). Studies show that a microfluidic ultrasonic method can fuse two kinds of membranes and simultaneously load drugs in the membrane 168. The poly (lactic-co-glycolic acid) (PLGA) NPs coated with EM and CCM were successfully prepared by this method. When it is difficult to modify the ligand directly on the EVs membrane, the membrane fusion method can be chosen to fuse the whole biofilm containing the required ligand with EVs to achieve the effect of surface modification 128.

## Application of Tumor-Derived Extracellular Vesicles in Cancer Therapy

The prepared drug-loaded EVs can realize the effect of targeted drug delivery. In particular, TDEVs can be used to target tumor cells to achieve information transfer, so the use of TDEVs based DDS has become a promising tumor treatment method. Among them, chemical drugs and biological drugs, especially natural products, can be delivered to the administration site through TDEVs, thereby enhancing the therapeutic effect of or reducing toxicity. Moreover, based on the direct encapsulation of simple compounds or biomolecules, some engineered drugs have also been designed to further optimize the construction of DDS 169, 170. This section discusses some representative studies (**Table 2**).

### Chemical Drugs

**Figure 6:**
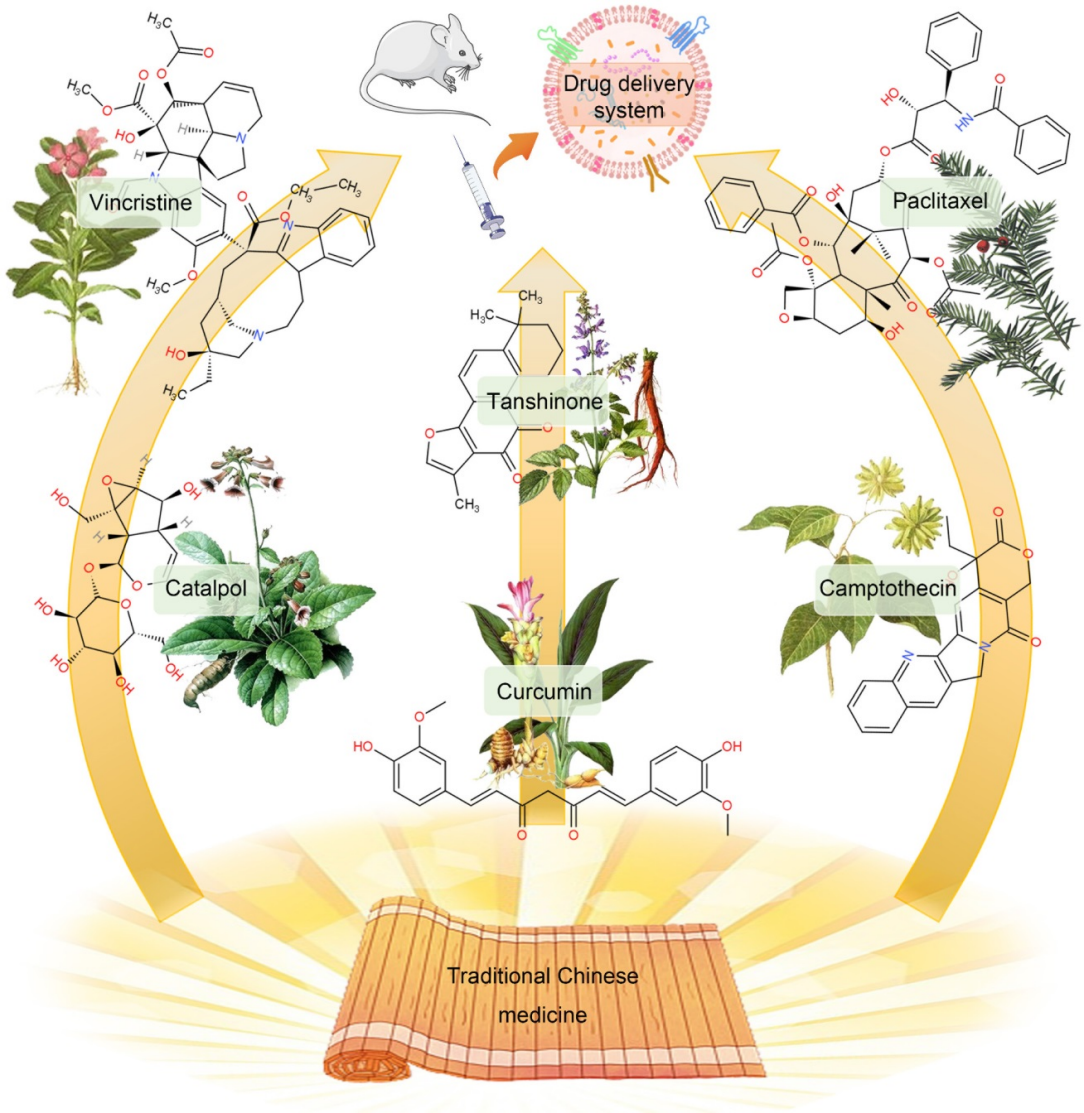
** The monomeric active ingredients in traditional Chinese medicine can be encapsulated in TDEVs to improve curative effects.** The construction of DDS with chemical drugs contained in TDEVs is a traditional therapy. However, some natural products with poor water solubility and low bioavailability, especially the monomer components of traditional Chinese medicine, can significantly improve the efficacy through the TDEVs delivery platform.

Since drug-loaded TDEVs carry tumor-specific antigens and other substances that can be efficiently taken up by tumor cells, they can improve the targeting effect of drugs and effectively reduce the viability of cancer cells. The traditional DDS construction is mainly through the packaging of simple chemical drugs into TDEVs. Among them, natural products are popular drugs of choice. In particular, many monomeric active ingredients are extracted from traditional Chinese medicines, such as paclitaxel (PTX), curcumin, camptothecin, vincristine, β-elemene, catalpol, tanshinone, and triptolide, etc. Most of these active ingredients of traditional Chinese medicine have the characteristics of high hydrophobicity, low solubility, poor stability, and short half-life. As a result, its bioavailability is low and it is difficult to be widely used in clinical practice. With the help of the administration of TDEVs, the curative effect of these Chinese medicine monomer components can be improved 171 (**Figure 6**).

PTX is a natural anti-tumor drug extracted from *Taxus chinensis*. It has poor water solubility, poor efficacy in intravenous injection, and has dose-dependent toxicity. These problems limit the use of PTX. Packing PTX with TDEVs can significantly improve its therapeutic effect. Saari *et al*. 131 isolated EVs from prostate cancer LNCaP and PC-3 PCa by DU. Flow cytometry and confocal microscopy demonstrated good uptake of the TDEVs by autologous prostate cancer cells, suggesting cancer cell targeting of the drug delivery system. EVs of 1×10^8^-5×10^9^ /mL were incubated in 1 mL of 5 μM PTX-DPBS for 1 h, loaded with PTX in TDEVs, and then centrifuged into balls. The loading rate was 9.2 ± 4.5%. The efficacy of the drug-loaded TDEVs was tested by bioassay. The results showed that the viability of LNCaP and PC-3 cells decreased by 80% and 40% respectively after 24 h administration. The mechanism of drug release is known as endocytosis.

Curcumin is one of the effective ingredients of the TCM *Curcuma wenyujin*. It has the effects of anti-inflammatory, anti-oxidative stress, and inhibiting the proliferation of malignant tumor cells. Curcumin can regulate different cancer factors without being toxic to normal cells. The National Cancer Institute has listed it as a third-generation cancer chemopreventive drug. Exosomes loaded with curcumin can increase its anti-tumor effect by 3-5 times. Co-culturing curcumin and pancreatic cancer cells can encapsulate curcumin in exosomes secreted by pancreatic cancer cells. By targeting pancreatic cancer cells, it can increase cytotoxicity. Studies have shown that after curcumin treats tumor cells, the secreted exosomes can inhibit IL-2 stimulated NK cell toxicity 172.

Camptothecin is a plant anti-cancer drug isolated from *Camptotheca acuminata*. It has a good effect on gastrointestinal as well as head and neck cancers. The exosomes secreted by colon-26 cells were loaded with camptothecin, and it was found that the killing effect on colon cancer cells was significantly higher than that of the free camptothecin group, the camptothecin exosomal mixed group, and the control group. It shows that the exosomes encapsulating camptothecin enhance the anti-cancer effect of camptothecin. In addition, a variety of TCM monomer components have been shown to be loaded by EVs to improve efficacy, but there are few reports on the use of TDEVs as a delivery platform 173.

TDEVs delivery of active ingredients of traditional Chinese medicine is expected to play a role in the treatment of a variety of tumors. One of the classic cases is the treatment of glioma. Glioma is highly malignant, has a poor prognosis, and is easy to relapse, which seriously threatens the safety of human life. At this stage, the clinical treatment of glioma is based on surgical treatment, radiotherapy, and chemotherapy. However, the infiltrating growth of glioma cells can easily cause radiation damage to the surrounding normal tissues. Surgical treatment is difficult to remove completely, and the side effects of radiotherapy and chemotherapy on patients are more obvious. A variety of active ingredients of traditional Chinese medicine show good glioma inhibitory effects. Including polyphenols, flavonoids, saponins, terpenes, alkaloids, and some mineral medicines containing arsenic, mainly natural products such as curcumin, resveratrol, silymarin, and quercetin. However, most of the drugs have problems such as poor solubility and poor stability, resulting in low bioavailability in the body after administration. In addition, due to the existence of the blood-brain barrier, the traditional Chinese medicine treatment of glioma still has problems such as poor drug targeting and many adverse reactions, resulting in poor therapeutic effects. In response to the above problems, in recent years, researchers have developed various drug delivery systems to increase the delivery of drugs in the brain. The targeted delivery system of traditional Chinese medicine can reach deep in the brain through blood circulation, increase the concentration and retention time of central nervous system drugs, improve the targeting efficiency of traditional Chinese medicine for glioma and reduce adverse reactions, and improve the therapeutic effect of glioma.

Traditional Chinese medicine has been increasingly used in the treatment of tumors due to its multi-targets and synergistic effects in recent years. The reports of EVs as emerging natural drug carriers have gradually increased, but there are still many biological problems need to be resolved. Compared with western medicine nano-preparations, the tumor therapeutic DDS prepared by encapsulating the effective ingredients of traditional Chinese medicine monomers does not show obvious advantages. The targeted delivery system of traditional Chinese medicine for the treatment of tumors still has some shortcomings. First, some drug delivery vehicles are easily recognized and eliminated by the immune system, which affects the targeting efficiency of drugs. Some carriers have a certain degree of biological toxicity, some carriers cannot be degraded in the organism, and there is a long-term hidden danger of toxicity to the organism. Moreover, the preparation methods of some vectors are complicated and cannot be mass-produced, and it is difficult for the Chinese medicine targeted delivery system to be transformed into clinical practice. In addition, there are few studies on the multi-component Chinese medicine compound targeted delivery system developed based on the theoretical characteristics of Chinese medicine to treat tumors. This kind of treatment method has not exerted the anti-tumor characteristics of Chinese medicine under the guidance of Chinese medicine theory. Therefore, the current research is mainly focused on the direction of TDEVs to deliver multiple components in the traditional Chinese medicine compound. Many of these problems have yet to be resolved, such as multi-component co-loading, coordinated delivery between components, and so on. At present, the reported preparations containing Chinese herbal compounds include Buyang Huanwu Decoction and Tongxinluo Capsules. However, DDS constructed by TDEVs containing Chinese herbal compounds has not been reported yet. However, with the continuous development of new materials, the continuous improvement of preparation processes, and the continuous development of carrier surface modifiers, the above-mentioned problems should be gradually solved. It is believed that the advantages of Chinese medicine targeted delivery system for the treatment of glioma will be fully reflected in the future.

The active ingredients in traditional Chinese medicine not only play a significant role as therapeutic drugs, but some traditional Chinese medicine ingredients themselves can exist as drug delivery carriers. Not only that, but traditional Chinese medicine also can produce EVs. At present, most researches focus on EVs derived from animal cells. In recent years, researches on EVs derived from Chinese medicine plants as active ingredients are gradually being carried out. Plant-derived EVs are easier to obtain in large quantities and the yield and economic benefits are greatly improved. They not only participate in the regulation of plant innate immune function, but also can perform the regulation of transboundary gene expression, achieve anti-inflammatory, anti-viral, and anti-oxidant effects, and provide new ideas for the study of active ingredients in traditional Chinese medicine. The active ingredients of traditional Chinese medicine and chemical drugs are used in combination, and they are jointly loaded in EVs, which can act on multiple signal pathways and play a synergistic effect. Play a slow and controlled release effect, increase drug loading, and reduce drug leakage while reversing tumor multi-drug resistance.

In addition to TCM ingredients, some antibiotics can also be used as encapsulated chemical drugs. DOX is one of the most effective anti-cancer drugs in clinical use. It has a significant inhibitory effect on many kinds of cancer cells. Due to its cardiac toxicity and other side effects, the dosage is limited 174. However, some structures in EVs, such as Cx43, have been shown to reduce cardiotoxicity and increase the release of EVs into tumor cells. Therefore, encapsulation of DOX in EVs is helpful to avoid its side effects, which can increase the accumulation concentration of the drug in the tumor and improve the therapeutic effect 176. Li *et al*. 134 isolated exosomes from A33-positive LIM1215 rectal cancer cells by ultracentrifugation and coated them with A33 antibody (A33Ab-US) on the surface of phase carboxyl superparamagnetic iron oxide nanoparticles (US). A33Ab-US-Exo/DOX was prepared by combining A33 on the exosome surface with an A33 antibody on the A33Ab-US surface. The encapsulation efficiency and loading capacities were about 9.06% and 2.60%, respectively. *In vivo* studies show that DDS can target A33 positive colon cancer cells, inhibit tumor growth, prolong the survival of mice, and reduce cardiac toxicity. Ingato *et al*. 175 prepared the NIbS/DOX nanovesicles induced by sulfhydryl blockade (NIbS) and by loading the chemotherapeutic drug into DOX nanovesicles. This vector is easy to be prepared on a large scale, and can significantly delay the growth of tumors and avoid non-specific distribution in important organs.

### Biological Drugs

In recent years, biopharmaceutical preparations have developed rapidly. Biopharmaceuticals are various regulatory substances related to metabolism, such as proteins, enzymes, nucleic acids, hormones, antibodies, cytokines, etc., extracted from the body. After the proper treatment, these biological drugs can also be incorporated into TDEVs to exert special effects.

Chemotherapeutic drugs and other biological drugs can be loaded into TDEVs to improve efficacy. Garofalo *et al*. 132 encapsulated the oncolytic virus (OVs) and PTX in the lung cancer cell-derived EVs as DDS for lung cancer treatment. OVs had an anti-cancer effect, and combined with PTX showed stronger cytotoxicity and oncolytic reaction. TDEV was collected from lung cancer cell A549 culture medium by ultracentrifugation. After incubating OVs and A549 cells with endogenous drug carriers, TDEVs containing OVs were obtained by centrifugation. Then, by a direct incubation procedure, TDEVs containing both OVs and PTX were obtained. *In vitro* and *in vivo* experiments showed that EV-virus-PTX significantly inhibited tumor growth. Garofalo *et al*. 133 prepared the OVs and PTX by the same method from the lung cancer cell LL/2 with the EVs package. The effect of DDS on the immune system was evaluated by generating a tumor model in immunocompetent homogenic mice (C57Bl/6). The results showed that the drugs were targeted at the tumor cells, and the OVs only caused inflammation around the tumor without affecting other parts of the body. OVs loaded into EVs alone can also play an anti-tumor role. Saari *et al*. 177 secrete the EVs by tumor cells infected with OVs by DGF and the virus was carried inside. The EVs can continue to infect other cells, achieving a tumor-suppressing effect.

**Figure 7:**
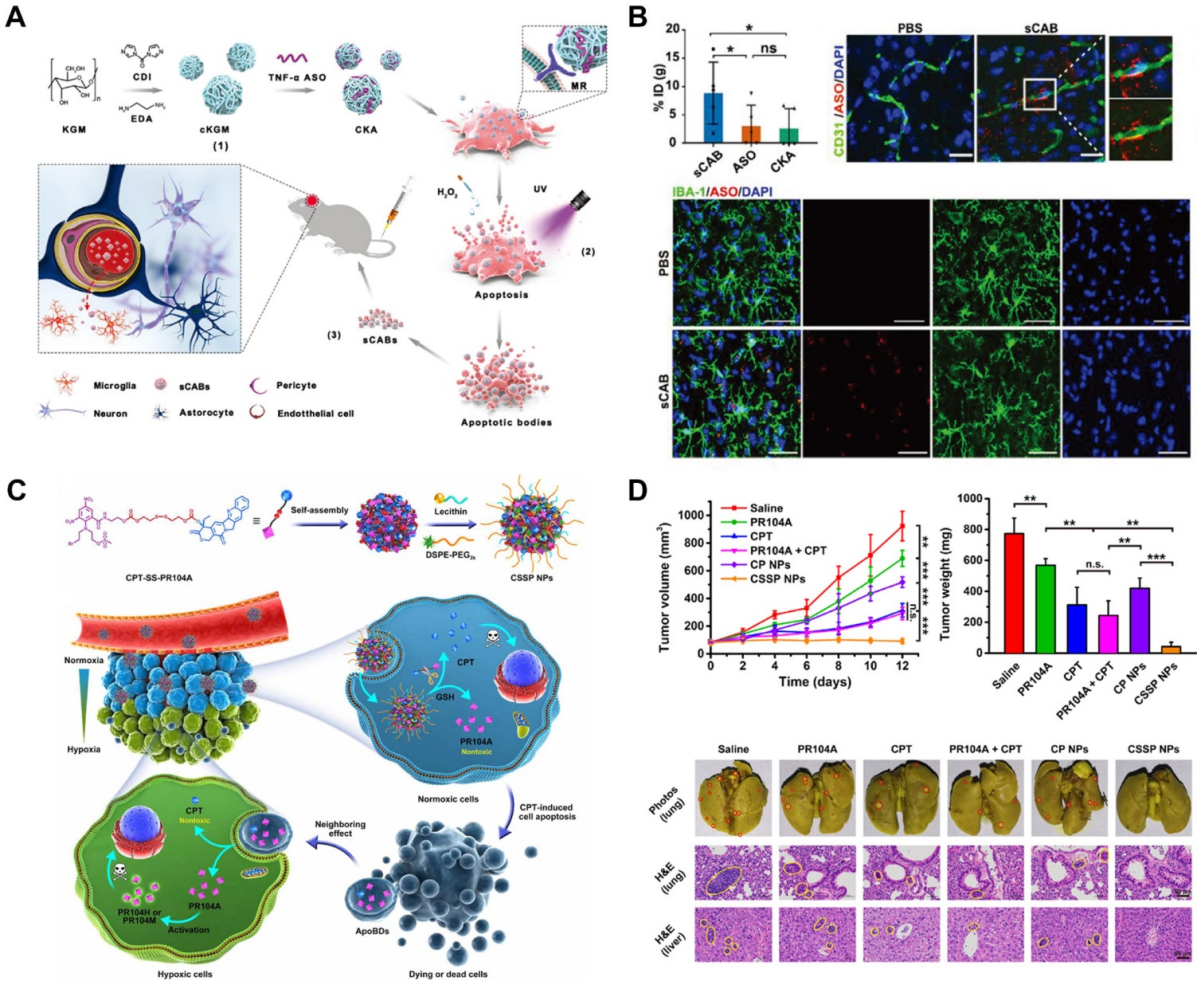
** Strategies for enhancing the effectiveness of cancer therapy.** A) Schematic graph of the convention for creating sCABs and conveying ASO into the cerebrum. B) Analysis of brain delivery efficiency, fluorescence microscopy images of a brain section, and the representative images from fluorescence microscopy. Adapted with permission from 181. Copyright Year 2021, Wiley-VCH GmbH. C) Manufacture of self-collected CSSP NPs. CSSP NPs upgrade drug entrance and entire cancer obliteration through the ApoBD-intervened adjoining impact. D) *In vivo* antimetastatic limit of CSSP NPs against orthotopic 4T1 tumors. Adapted with permission from 191. Copyright Year 2021, AAAS.

Gene therapy is an important method of cancer treatment. Gene therapy can directly regulate the growth of tumor cells, but also can activate the body's immune system to eliminate cancer cells. However, the low absorptivity and high cytotoxicity of gene drugs limit their delivery *in vivo*, resulting in low clinical efficacy. Therefore, it may be considered to load the programmable RNA drug into the low immunogenicity TDEVS and deliver it directly to tumor cells to improve the therapeutic effect 178-180. Genetic drugs are often loaded into TDEVs as plasmids. Kim *et al*. 139 secreted the exosome by ovarian cancer cell line SKOV3 using ExoQuick^™^. Expression of CRISPR/Cas9 plasmid in exosomes by electroporation. The expression of poly (ADP-ribose) polymerase-1(PARP-1) was inhibited by the exosomes loaded with CRISPR/Cas9, which activated the apoptotic pathway and inhibited the proliferation of ovarian cancer cells. Moreover, compared with the exosomes derived from epithelial cells, they are more targeted. Wang *et al*. 181 combined cationic konjac glucomannan (cKGM) with anti-TNF-α antisense oligonucleotide. Load drugs in the apoptotic bodies (sABs) of brain metastatic cancer cells. The DDS can penetrate the blood-brain barrier mediated by CD44v6 and be absorbed by the brain microglia (**Figure 7A-B**).

Tumor immunity, as a common cancer treatment strategy, has received more and more attention in recent years. Cancer cells constantly express new tumor antigens that normal cells do not possess 182, 183. Under normal circumstances, the immune system recognizes the new antigens and initiates a series of immune events to clear out the cancerous cells. First, new antigens produced by cancer cells are captured by antigen-presenting cells, such as dendritic cells (DCs), and processed into a major histocompatibility complex for downstream cell recognition. Subsequently, T lymphocyte recognition, initiation of specific antigen response, invasion of tumor cells, T lymphocyte receptor (TCR) binding to MHC-Ⅰ, clearance of cancer cells. The dead cancer cells release the antigen again for DCs to identify and complete the immune cycle. Some drugs boost the body's immune response to tumor cells, so they can be delivered via TDEVs. However, some studies show that TDEVS can promote tumor immune escape, so whether drug-loaded TDEVs can achieve the therapeutic effect needs further experiments. In the treatment of tumors, focused ultrasound heat can affect the release of TDEVs and promote innate immune activation 184-187. Cheng *et al*. 188 achieved the synthesis of multivalent antibody-retargeting exosomes (SMART-Exos) that express T cell CD3 and cancer-associated EGFR monoclonal antibody not only induce T cells to cross-link with EGFR-expressing breast cancer cells and can induce strong anti-tumor immunity *in vivo*. TDEVs loaded with some special kinds of miRNAs can modulate the immune response and tumor microenvironment. Then the DDS can initiate the anti-tumor response. Taghikhani *et al*. 140 isolated exosomes from breast cancer cell lines and electroporation loaded TDEV with miR155, miR142, and let7i enhanced tumor immunity and induced potent dendritic cell. Asadirad *et al.* 189 similarly loaded miRNA-155 by electroporation onto tumor cell-derived exosomes to assess the stimulation of the dendritic cell.

### Engineered drugs

Past studies usually directly load chemotherapeutic drugs or biomolecules in TDEVs. However, in recent years, more and more researchers tend to combine or modify drugs and then deliver them through TDEVs to improve the therapeutic effect. This process usually involves the recombination of multiple drugs. For example, simple active molecules can be first made into biomimetic nanoparticles 190. Yong *et al*. 135 designed a kind of biocompatibility extracellular endocytic biomimetic porous silicon nanoparticle (PSINPS). Human liver cancer Bel7402 cells were incubated with endogenous drug-loaded PSINPS and then centrifuged to collect drug-loaded exosomes. A DOX-loaded PSiNPs (DOX@E-PSiNPs) can be obtained from the ectosomal sheath. The DDS demonstrated significant cell take-up and cytotoxicity in both cancer cells and cancer stem cells (CSCs). It improved cancer aggregation, extravasation of veins, and infiltration of the growth profound parenchyma.

DDS based on apoptotic bodies is also an effective cancer therapy and can enhance combined chemical drugs penetration. Zhao *et al*. 191 demonstrated that apoptotic bodies can carry residual drugs to adjacent tumor cells after apoptosis. In this study, camptothecin (CPT) and prodrug PR104A were combined to form disulfide nanoparticles, in which CPT can kill external oxygenic tumor cells and produce apoptotic bodies. The apoptotic bodies then transport the remaining drugs into the tumor, and PR104A is activated to exert a strong anti-tumor effect. Apoptotic bodies play a special role in drug delivery and facilitate drug penetration, overcoming the limitation of proximity effect in penetrating solid tumors (**Figure 7C-D**). In the above example, EVs are not isolated from the tumor, which opens a new way for TDEVs based DDS fabrication. Other studies have used DDS with a similar structure to EVs to improve efficacy.

**Figure 8:**
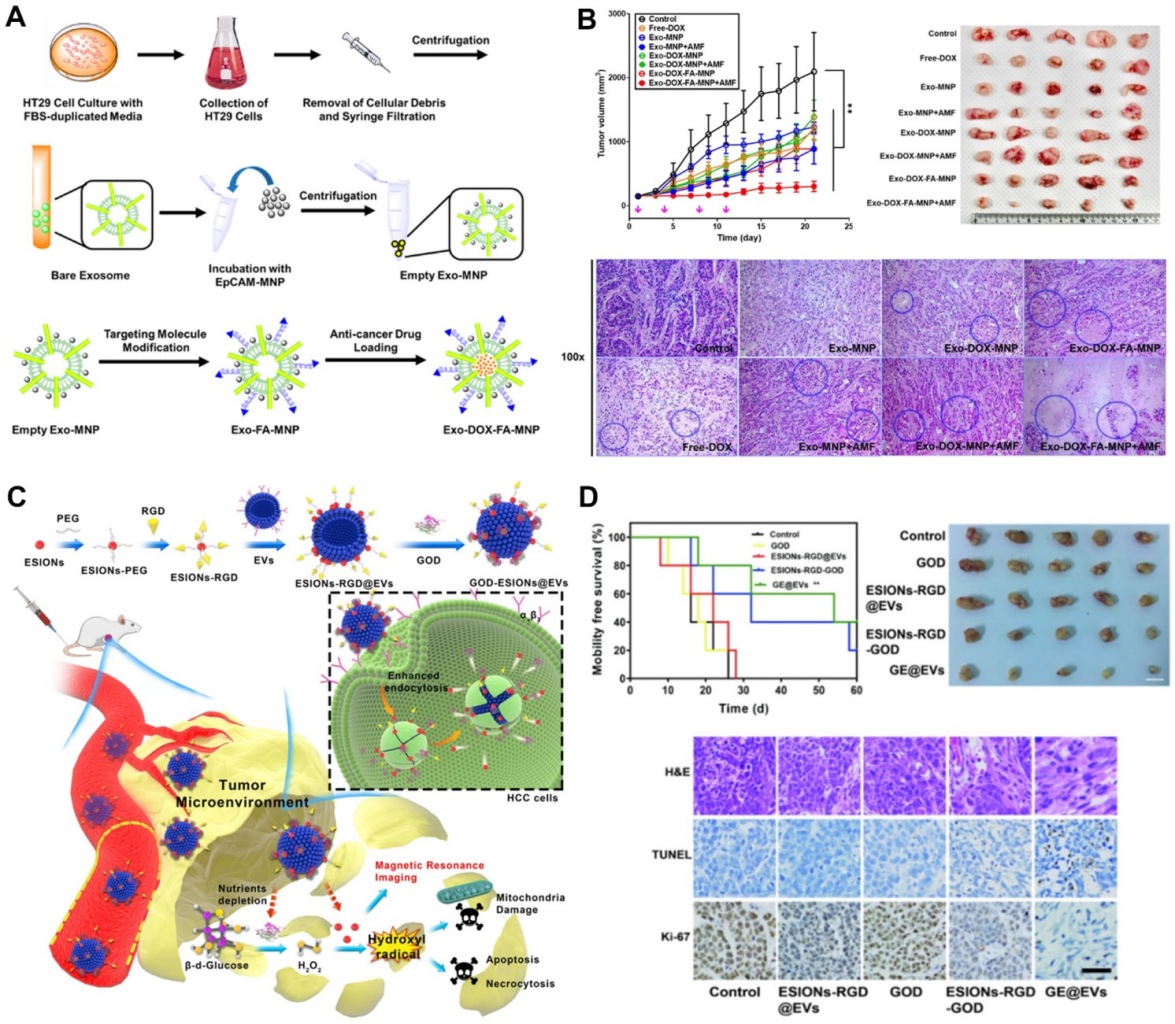
** New drugs that can be used to make DDS.** A) Exosome-based cross breed nanostructure readiness and portrayal. B) Tumor sizes in various trial gatherings. Cancer weight changes in various trial gatherings. *In vivo* cancer tissue investigations. Adapted with permission from 192. Copyright Year 2021, Elsevier. C) Schematic outline of the manufactured course of GE@EVs and successive reactant treatment against HCC with improved intracellular endocytosis. D) *In vivo* diagnostic imaging and remedial viability for HCC tumor-bearing mice. Adapted with permission from 193. Copyright Year 2021, IVYSPRING.

There are also new drugs that can be used to make DDS. The emerging nano-catalytic drugs have become an alternative to chemotherapeutic drugs. Wu et* al*. 192 secreted EVs derived from hepatocellular carcinoma (HCC) cells by ultracentrifugation. EVs were then mixed with extremely small-sized iron oxide particles (ESIONS)-RGD solution for 24 h. Then mixed the EVs with glucose oxidase (GOD) for 24 h. Finally, EVs were loaded with GOD and ESIONS, and a tumor-specific and cascaded nano-catalytic therapy GOD-ESIONs@EVs (GE@EVs) for HCC was successfully prepared. Continuous nanocatalyst prolongs the duration of DDS therapy in the tumor region through arginine-glycine-L-aspartic acid-targeting and membrane fusion to enhance endocytosis. In addition, Kwon *et al*. 193 loaded DOX into EVs and surface modification with magnetic nanoparticles (MNPs) and tumor-targeting ligand folate. Both chemotherapy and hyperthermia can be achieved. The results showed that colorectal cancer could be inhibited by increasing the targeting ability. The DDS is relied upon to beat the restrictions of the current framework as a powerful medication conveyance approach for malignancy treatment (**Figure 8**).

**Figure 9:**
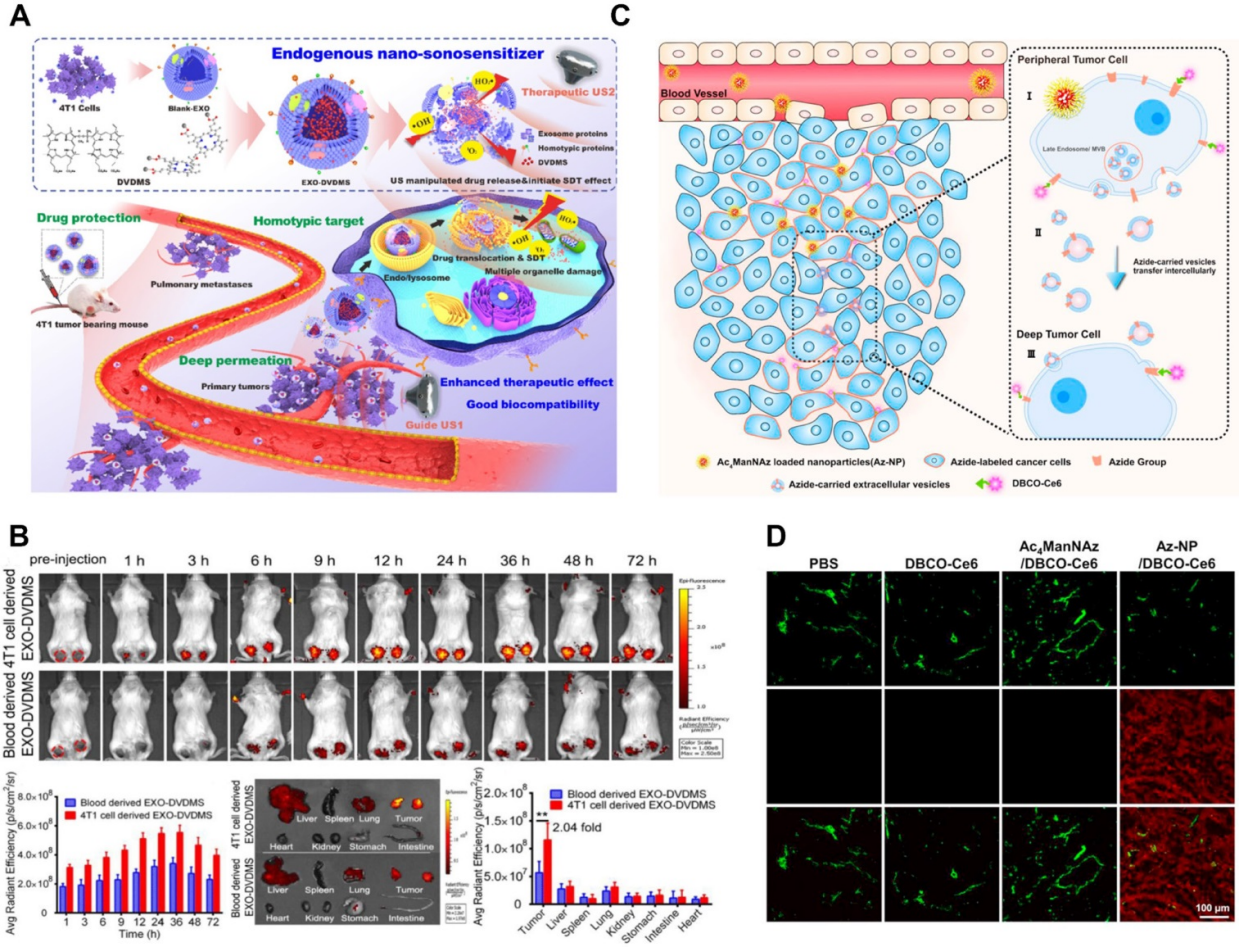
** Other strategies for tumor-derived extracellular vesicles in cancer therapy.** A) Outline of endogenous nanosonosensitizers for centered US-expanded focusing on conveyance to perceive homotypic malignant growth cells, boosts responsive medication discharge, and improved SDT. B) *In vivo* homotypic targeting potential of EXO-DVDMS derived from 4T1 tumor cells. Includes *in vivo* images, quantification, *ex vivo* images, and quantitative analysis of fluorescence intensity. Adapted with permission from 138. Copyright Year 2019, IVYSPRING. C) Schematics of nanoparticle-intervened metabolic marking of azide groups on the perivascular tumor cell film and intercellular exchange of the azide gatherings to profound cancer cells through EVs for upgraded cancer focusing on and infiltration of DBCO-Ce6 by a bioorthogonal click reaction. D) Confocal pictures of frozen areas of tumors that were extracted from mice after treatment with DBCO-Ce6, Ac_4_ManNAz/DBCO-Ce6, or Az-NPs/DBCO-Ce6. Adapted with permission from 197. Copyright Year 2020, Elsevier.

Not only are the principles of passive and active targeting applied to the construction of DDS, but the strategy of physical chemistry can also realize the targeting effect of drug-loaded TDEVs. This principle can also achieve good results 194, 195. Liu *et al*. 138 extracted exosomes from mouse breast cancer 4T1 cells by ultracentrifugation. Then incubated them with DVDMS to prepare a functional intelligent nanosensitizer (exo-DVDMS). The drug carrier can be swallowed by lysosomes, trigger DVDMS relocalization under low pH and ultrasound conditions. It can initiate multiple cell death signaling pathways and be used for ultrasound response controlled release and enhanced sonodynamic therapy (**Figure 9A-B**).

Some studies have produced EVs that target hypoxic tumor cells, for example, to enhance tumor response 196. Jung *et al*. 136 used the iron chelator desferrioxamine (DFO), which can up-regulate the expression of hypoxia-inducible factors 1a (HIF-1a) and increase the expression of the HIF-1a target gene associated with hypoxia. Human breast cancer cell line MDA-MB-231 cells were cultured in a humidified environment at 37ºC and 5% carbon dioxide for 24 h, simulating hypoxia. Exosomes were extracted from anoxic cells by ExoQuick^™^ kit and then loaded with Olaparib (PARP inhibitor) by electroporation and labeled with superparamagnetic iron oxide (SPIO). Compared with other DDS, hypoxic cells preferentially absorb the exosomes released by hypoxic cells, increase cell apoptosis and slow down tumor growth *in vivo*. Zhu *et al*. 137 extracted EVs from mouse breast cancer cell 4T1 by commercial kit, and macrosperm exocrine/AIEgen hybrid nanovesicles (called DES) were prepared by electroporation. The DDS can promote the deep penetration of the tumor. Moreover, the use of dexamethasone can reduce local hypoxia in tumors and improve efficacy. Tu *et al*. 197 enhanced tumor targeting and penetration by the synthesis of nanoparticles-mediated metabolic tumor ligands. First, artificial azide ligands are labeled on tumor cells around blood vessels. These tumor cells will release TDEVs. The azide ligands are passed from cell to cell and eventually taken up by tumor cells deep within the tumor. The ligands are then transferred to the deep tumor. A highly selective *in vivo* bioorthogonal click-response to azide on the surface of deep tumors was observed by intravenous injection of a water-soluble dibenzo cyclooctyne modified chloride e6 (DBCO-Ce6). Through the strategy of DDS construction, the accumulation and penetration of DBCO-Ce6 in the tumor were enhanced (**Figure 9C-D**).

## Clinical Trials Progress and Challenge of Extracellular Vesicles as Drug Delivery System

The EVs-based cancer therapy drug delivery system has many advantages. Specifically, EVs can prolong the half-life of drugs to maintain stable drug properties. Moreover, EVs have good biocompatibility, which makes the drugs easier to be absorbed by the body and avoids suffering from drug resistance. In particular, TDEVs can better recognize tumors and contain tumor-associated antigens and immunosuppressive molecules, which can down-regulate the immune system's response and deliver certain inhibitory signals. Therefore, DDS based on TDEVs can bring better therapeutic effects to a certain extent. The above-mentioned advantages have made the research of EVs as drug carriers attract more and more attention in recent years 2, 5, 198.

However, there are also many noteworthy problems in the clinical application of the DDS. First, to improve the efficiency of EVs administration, appropriate cell sources should be selected, taking full account of tumor cells and cells in the tumor microenvironment. The selection of parental cells not only needs to consider factors such as the activity of EVs, tissue homing ability, immunogenicity, and carcinogenicity but also whether the parental cells are convenient for large-scale culture. In addition, the parental cells should have the characteristics of genetic stability and fewer pollutants. In the process of large-scale cultivation, genetic stability and the influence of pollutants should also be closely monitored. Second, appropriate extraction methods and drug loading methods should be selected. The EV concentration is low, the content is small, and there are complex environmental components. If there are more impurities in EVs, it may affect the quality of the preparation, so sufficient purification should be given to ensure the removal of impurities, such as cell debris. When selecting an extraction method, comprehensive consideration should be given to the yield, purity, integrity, and efficiency of EVs, and whether the method can be mass-produced. The drug delivery method should be cost-effective and efficient. The design of the EVs specific drug delivery program is a cautious process, and comprehensive experimental verification is required to determine a EVs preparation that can achieve large-scale clinical production. When performing operations such as destroying the membrane during the drug loading process, there is a risk of changing the direction of the membrane protein, which may cause recognition by the immune system and then cause adverse reactions. When constructing DDS, the targeting of TDEVs may be insufficient, and surface modification is needed to improve the effectiveness of targeted therapy. Third, the storage of EVs is also a problem that should be paid attention to. Higher temperatures may change the physical and chemical properties of the vesicles and reduce the effectiveness of the load. For example, storage of exosomes at 4°C will cause aggregation and structural destruction. The current better storage condition is less than -80°C. However, this storage condition also increases the cost of transportation and storage. It is worth noting that there are still many controversies about the *in vivo* function and safety of EVs. In the clinical use of EVs for drug delivery, potential risks need to be considered. Since TDEVs play a role in assisting cancer cells to build the local and distant microenvironment of the tumor during the tumorigenesis and development process, these DDS can not only deliver drugs but also increase the proliferation and spread of tumor cells in the body. In addition, because EVs can carry drugs across biological barriers, it may increase the dose of drugs in certain special tissues and organs, leading to increased toxicity. Moreover, the cell-to-cell communication of endogenous EVs may be interrupted due to the influence of a large number of exogenous EVs. The protein on the surface of the vesicle may cause biological toxic side effects. In *in vivo* and *in vitro* studies, how to determine the final active substance, inactive ingredients, and mode of action is also a challenge. Therefore, when applying EVs-based DDS, it is necessary to consider selecting EVs derived from appropriate cells and tissues, as well as appropriate drugs and modifying them with other compounds to avoid the occurrence of the above-mentioned situations (**Table 3**).

Since more than 10 years ago, many pharmaceutical companies have announced the development of therapeutic EVs as drug delivery vehicles. The clinical research of EVs on cancer treatment began to rise, gradually transforming from scientific research results to applications, and the safety and effectiveness of the technology have been verified. Drug-loaded EVs have been applied to the treatment of a variety of cancers, such as prostate cancer, brain cancer, lung cancer, bowel cancer, melanoma, and so on. At present, the clinical transformation of most EVs on a global scale has progressed to preclinical or clinical phase I, and some have entered clinical phase II. Most clinical trials have shown good efficacy. In recent years, some biotech companies have listed several EVs drug delivery systems. Aegle Therapeutics is the first company approved by the FDA to enter human clinical trials. Founded in 2015, CODIAK is a biotechnology company focusing on cancer immunity. The candidate drug exoIL-12^TM^ has entered clinical phase I and can treat skin T-cell lymphoma, melanoma, and breast cancer. The clinical trials showed positive results and demonstrated good safety. exoSTING^TM^ can treat solid tumors such as the neck, head squamous cell carcinoma, and breast cancer, and has entered phase I/II trials. Other drugs such as exoASO-STAT6 and exoASO-STAT3 are in the preclinical stage. Evox Therapeutics, a British biotechnology company dedicated to engineering exosomes for delivery, raised US$95.4 million to advance its exosome therapy into clinical trials. MD Anderson also initiated the first clinical trial of therapeutic exosomes. At present, China's innovative "drug-loaded vesicle therapy technology" has completed the clinical transformation in the Chinese market and has been approved for use in 7 provinces and cities in Hubei, Hunan, Hebei, Shandong, Anhui, Shenzhen, and Tianjin, and has become EVs research and clinical transformation The most advanced new tumor treatment technology. In the process of clinical transformation of EVs, the biological research, technology, and application of EVs are closely related and complement each other. The development of production technology relies on a deeper understanding of the biological research of EVs, and clinical transformation is inseparable from the innovation of production technology 6-8, 199, 200.

In summary, the application prospects of EVs are full of hope. Even if many unsolved problems require further research by experimenters, if DDS based on EVs can effectively achieve targeted delivery, a new era of tumor therapy will come. This technology will fill the gaps in multiple tumor treatment methods and bring the gospel to more cancer patients. It is believed that EVs will bring new disease treatment opportunities in the near future.

## Figures and Tables

**Table 1:** Drug loading methods in EVs.

EVs sources	Loading content	Loading method	Loading results	Ref.
Prostste cancer cell (LNCaP and PC-3 PCa)	PTX	Incubate 1×10^8^-5×10^9^ EVs/mL in 1 mL of 5 μM PTX-DPBS solution for 1 h at 22ºC.	Loading efficiency is 9.2 ± 4.5%.	131
Human lung cancer cell (A549)	PTX and oncolytic adenovirus	Incubation with mixing for 1 h and carried out at RT. Samples were then centrifuged at 150,000 × g for 2 h at RT, to pellet EV-Virus-PTX.	The UPLC assessed concentration of the PTX control sample shows a 38% loss of PTX.	132
Mouse lung cancer cell (LL/2)	PTX and oncolytic adenovirus	For *in vitro* samples, incubating 1 × 10^8^-5 × 10^9^ EVs in 1 mL of 5 μM PTX-PBS solution, and 10 μM PTX-PBS solution for *in vivo* samples, for 1 h at 22ºC.	The Cryo-EM images demonstrated that the free virus was less frequent compared to the encapsulated virus.	133
Human CRC cell line (LIM1215)	DOX	Mix 200 μg exosomes with 20 μg DOX for 5 min dialyzed overnight in PBS. This followed with mixing with A33Ab-US overnight at 4ºC at the optimal proportion.	The DOX encapsulation efficiency and loading capacities were about 9.06% and 2.60%.	134
Human hepatocarcinoma cell line (Bel7402)	PSiNPs with DOX inside	After 16 h incubation, the debris was discarded at 5,000 g for 15 min and then the supernatants were further centrifuged at 20,000 g for 30 min.	It can strongly be confirmed that the membrane that sheathed PSiNPs in E-PSiNPs is exosomes.	135
Human breast cancer cell line (MDA-MB-231)	Olaparib (PARP inhibitor)	Electroporation was performed at 150 mF and 350 V using a Gene Pulser Xcell Electroporatoin System (Bio-Rad, Hercules, CA, USA) in electroporation cuvettes.	The size ranging from 30 to 200 nm diameter.	136
Mouse breast cancer cell (4T1)	Dexamethasone	Electroporation	The particles were < 500 nm in size, and electroporation didn't fundamentally adjust the morphology of these particles.	137
Mouse breast cancer cell (4T1)	Sinoporphyrin sodium (DVDMS)	DVDMS and 1 μg/μL exosomes were mixed in different ratios (1:30, 1:15.5, 1:7.5, 1:3), and incubated for 30 min at room temperature.	Loading efficiency is 5.18%.	138
Ovarian cancer cell line (SKOV3)	Cas9-/sgRNA-expressing plasmids	30 μg of exosomes was mixed with 10 μg of DNA in R buffer from the Neon kit (Invitrogen) before electroporation. After electroporation, exosomes were washed a few times with PBS.	Loading efficiency is 1.75%.	139
Mouse breast cancer cell (4T1)	miR‐155, miR‐142, and let‐7i	Electroporation	In the aforementioned groups, the level of mir‐155, miR‐142, and let7i increased to 31.47, 47.2, and 44.13‐fold respectively in modified TEXs	140

Abbreviations: CRC: colorectal cancer; DOX: doxorubicin; PTX: paclitaxel; RT: room temperature; UPLC: ultra performance liquid chromatography.

**Table 2:** Studies on the use of TDEVs as drug delivery systems in cancer treatment.

EVs sources	Durg	Disease models	EVs isolation	Loading	Engineering	Results	Ref.
Prostste cancer cell (LNCaP and PC-3 PCa)	PTX	Prostate cancer	Differential ultracentrifugation	Incubation	None	TDEVs can be utilized as viable transporters of PTX to their parental cells. They carry the drug into the cells through an endocytic pathway, so it can increase its cytotoxicity.	131
Human lung cancer cell (A549)	PTX and oncolytic adenovirus	Human lung cancer	Differential ultracentrifugation	Incubation	None	Joined therapy of OVs and PTX encapsulated in EV has improved anticancer impacts both *in vitro* and *in vivo* in lung cancer models.	132
Mouse lung cancer cell (LL/2)	PTX and oncolytic adenovirus	Lung cancer	Ultracentrifugation	Incubation	None	This study emphatically supports the fundamental organization of EVs formulations with OVs alone or in blend with chemotherapy agents as a novel strategy pointed toward treating essential and metastatic malignant growths.	133
Human CRC cell line (LIM1215)	DOX	Colon cancer	Ultracentrifugation	Incubation	A33Ab-US	*In vivo* study showed that A33Ab-US-Exo/DOX had astounding cancer focusing on capacity, and had the option to restrain cancer development and drag out the endurance of the mice with decreased cardiotoxicity.	134
Human hepatocarcinoma cell line (Bel7402)	PSiNPs with DOX inside	Hepatocarcinoma	Ultracentrifugation	Endogenous drug loading	None	These properties endow DOX@E-PSiNPs with extraordinary *in vivo* enrichment of incomplete cancer cells and side populace cells with provisions of CSCs, bringing about anticancer action and CSCs decrease in orthotopic, subcutaneous, and metastatic tumor models.	135
Human breast cancer cell line (MDA-MB-231)	Olaparib (PARP inhibitor)	Breast cancer	ExoQuick^™^	Electroporation	SPIO remark	This novel theranostic stage can be used as a compelling system to screen exosomes *in vivo* and convey therapeutics to hypoxic tumors.	136
Mouse breast cancer cell (4T1)	Dexamethasone	Breast cancer	Commercial isolation kit	Electroporation	None	It accomplished the hybridization of AIEgen and biological tumor-exocytosed exosomes interestingly, and join PDT approaches with normalizing the intratumoral vasculature as a method for lessening nearby tissue hypoxia.	137
Mouse breast cancer cell (4T1)	Sinoporphyrin sodium (DVDMS)	Breast cancer	Ultracentrifugation	Incubation	Ultrasound-responsive	The exosomal detailing filled in as a functionalized nanostructure and worked with concurrent imaging and cancer metastasis restraint, which were respectively 3-folds and 10-folds higher than that of free form.	138
Ovarian cancer cell line (SKOV3)	Cas9-/sgRNA-expressing plasmids	Ovarian cancer	ExoQuick^™^	Electroporation	None	The hindrance of PARP-1 by CRISPR/Cas9-mediated genome altering upgrades the chemosensitivity to cisplatin, showing synergistic cytotoxicity.	139
Mouse breast cancer cell (4T1)	miR‐155, miR‐142, and let‐7i	Breast cancer	Differential ultracentrifugation	Electroporation	None	It enhances the immune stimulation ability and induces potent DCs.	140

Abbreviations: CSCs: cancer stem cells; DOX: doxorubicin; OVs: oncolytic virus; PTX: paclitaxel.

**Table 3:** Challenges of TDEVs in constructing drug-carrying systems.

Aspects	Challenges	Solutions
Cell culture	Large-scale cultivation costs are high.	Choose the appropriate parental cell and culture method 5.
	There are many impurities in the culture solution that are not easy to separate 2.	Closely monitor gene stability and the influence of contaminants during the cultivation process.
Method of isolation	The biological environment in which TDEVs exist may contain other impurities, such as cell debris, which affect purity. Further interfere with the determination, or cause the quality of the preparation to decrease 6.	Should be extracted multiple times, or combined with different methods of extraction and purification. Try to purify to remove interfering substances.
	The content of TDEVs is small and the concentration is low. It takes more cost to extract the required amount 7.	Improve the efficiency of the extraction method and choose a method with a relatively high sensitivity.
	Excessive operation time or some extraction methods based on the surface components of TDEVs may destroy the integrity of TDEVs.	Choose the appropriate method according to the needs of DDS construction, sample type, impurity type, etc 8.
Drug loading method	The drug loading process involves damage to the vesicle membrane, or changes in certain physical and chemical properties, which may affect the safety of the preparation 198.	During the drug loading process, attention should be paid to changes in membrane proteins to avoid causing immune reactions. Conduct comprehensive clinical trials to ensure its safety.
	Due to the biological function of TDEVs in cancer progression, it may promote the occurrence and development of tumors.	Design a DDS whose therapeutic effect is greater than that of cancer promotion, and conduct experiments to verify that the DDS is mainly therapeutic.
Transport and storage	Higher temperatures may cause structural damage to the vesicles and reduce the effective load 199.	Store below -80℃.
Targeting	Insufficient targeting, the accumulation of drugs at the tumor site cannot reach an effective concentration.	Fully consider factors such as tumor cells and cell activity in the tumor microenvironment, tissue homing ability, immunogenicity, and carcinogenicity, and select appropriate parental cells. In addition, surface modification can also improve the targeting of DDS.
	The protective effect of biological barriers.	Construct DDS that can pass through biological barriers and overcome its hindrance in drug delivery. However, care should be taken to avoid its accumulation in the barrier and produce toxic side effects 200.
	There is an accumulation of drugs in non-tumor areas.	Choose appropriate drugs and DDS construction methods to reduce side effects.
	The introduction of a large number of exogenous EVs may affect the signal transduction function of endogenous EVs, leading to disorder of information transmission.	Conduct comprehensive clinical trials to ensure its safety.

## Abbreviations

ABC: ATP binding cassette
ABs: apoptotic bodies
AF4: Asymmetric flow field-flow fractionation
BBB: blood-brain barrier
BCRP: breast cancer resistant protein
CAFs: cancer-associated fibroblasts
CCM: cancer cell membrane
CPP: cytopenetrating peptide
CRC: colorectal cancer
CSCs: cancer stem cells
CSPG4: chondroitin sulfate proteoglycan 4
CTC: circulating tumor cells
DC: dendritic cells
DDS: drug delivery systems
DGF: density gradient flotation
DOX: doxorubicin
DU: differential ultracentrifugation
ECM: extracellular matrix
ECs: endothelial cells
EGFR: epidermal growth factor receptor
EM: exosomal membrane
ESCRTs: endosomal sorting complex required for transport
EVs: extracellular vesicles
FFFF: Flow field-flow fractionation
GPCR: G protein-coupled receptor
GPI-Aps: glycosylphosphatidy-linositol-anchored protein
HA: hyaluronic acid
HBV: hepatitis type B
HCC: hepatocellular carcinoma
IHH: indian hedgehog homologs
ILVs: intraluminal vesicles
MDSCs: myeloid-derived suppressor cells
MLC: myosin light chain
MMP: matrix metalloproteinases
MRP: multidrug resistance-associated protein
MVBs: multivesicular bodies
MVs: microvesicles
NK: natural killer
NPs: nanoparticles
NSCLC: non-small-cell lung carcinoma
OC: ovarian cancer
OVs: oncolytic virus
PEG: polyethylene glycol
PLD: PEGylated liposomal doxorubicin
PLGA: poly (lactic-co-glycolic acid)
PMN: pre-metastatic niche
PTX: paclitaxel
ROCK-1: Rho-associated protein kinase 1
RT: room temperature
RVG: rabies virus glycoprotein
SEC: size exclusion chromatography
sEV: small extracellular vesicles
SNAREs: *N*-ethylmaleimide-sensitive fusion protein attachment protein receptor
TCM: traditional Chinese medicine
TDEVs: tumor-derived extracellular vesicles
Tf-TfR: transferrin-transferrin receptor
TME: tumor microenvironment
TNF-R1: tumor necrosis factor receptor 1
TRAIL: TNF-related apoptosis-inducing ligand
UPLC: ultra-performance liquid chromatography
